# The current status and further prospects for lung magnetic resonance imaging in pediatric radiology

**DOI:** 10.1007/s00247-019-04594-z

**Published:** 2020-01-29

**Authors:** Franz Wolfgang Hirsch, Ina Sorge, Jens Vogel-Claussen, Christian Roth, Daniel Gräfe, Anne Päts, Andreas Voskrebenzev, Rebecca Marie Anders

**Affiliations:** 1 Department of Pediatric Radiology, University of Leipzig, Liebigstraße 20a, 04103 Leipzig, Germany; 2 Institute for Diagnostic and Interventional Radiology, Hannover Medical School, 30625 Hannover, Germany; 3 Biomedical Research in End-stage and Obstructive Lung Disease Hannover (BREATH), German Centre for Lung Research, 30625 Hannover, Germany

**Keywords:** Children, Lung, Magnetic resonance imaging, Ultrashort echo time

## Abstract

Lung MRI makes it possible to replace up to 90% of CT examinations with radiation-free magnetic resonance diagnostics of the lungs without suffering any diagnostic loss. The individual radiation exposure can thus be relevantly reduced. This applies in particular to children who repeatedly require sectional imaging of the lung, e.g., in tumor surveillance or in chronic lung diseases such as cystic fibrosis. In this paper we discuss various factors that favor the establishment of lung MRI in the clinical setting. Among the many sequences proposed for lung imaging, respiration-triggered T2-W turbo spin-echo (TSE) sequences have been established as a good standard for children. Additional sequences are mostly dispensable. The most important pulmonary findings are demonstrated here in the form of a detailed pictorial essay. T1-weighted gradient echo sequences with ultrashort echo time are a new option. These sequences anticipate signal loss in the lung and deliver CT-like images with high spatial resolution. When using self-gated T1-W ultrashort echo time 3-D sequences that acquire iso-voxel geometry in the sub-millimeter range, secondary reconstructions are possible.

**Electronic supplementary material:**

The online version of this article (10.1007/s00247-019-04594-z) contains supplementary material, which is available to authorized users.

## Introduction

Many children need regular lung imaging. Developments in MRI make this a viable radiation-free alternative for pediatric chest imaging. Special considerations in pediatrics have led to the development of these techniques, which could subsequently influence adult practice.

In principle, a morphological quality standard in MRI has been attainable for several years, and this in many cases is in no way inferior to pulmonary CT [1]. In addition, lung MRI offers the possibility of functional assessment regarding ventilation and perfusion [2], which can only be achieved with functional diagnostics or nuclear medical methods at this point. Functional lung MRI does not yet play a role in clinical routine [3, 4]. The exclusively morphological evaluation of the lung with conventional MR proton imaging has become the method of first choice at many pediatric radiology facilities when sectional imaging of the lung is necessary [5, 6].

Although the radiation exposure of the thoracic CT with new CT detector technology is really fairly low, any radiation exposure to children should be avoided if possible because radiation-sensitive organs (breasts, thyroid gland) are exposed. In addition, the tissue differentiation of some lung processes is better possible with MRI than CT. This is shown later in this pictorial review. The use of gadolinium (which some people have reservations against) is usually not necessary in lung MRI but makes co-evaluation of the mediastinum possible, whereas in CT a contrast medium has to be used.

In this paper we discuss what is done better — or differently — at hospitals that incorporate lung MRI from institutions that continue to perform only pulmonary CT. Technical aspects of lung imaging are therefore discussed. Subsequently, representative morphological lung MRI examinations are shown in this pictorial essay. The image quality presented, which is now standard, is likely to convince even skeptics of the diagnostic potency of lung MRI.

## Why magnetic resonance imaging?

Only if you succeed in convincing the clinical partners of the unfamiliar image impression of the MRI and if you succeed in gaining understanding for the fact that about 10% of MRIs of the lung are diagnostically insufficient, will the chest MRI become established as routine lung diagnostics. The main point for the use of pulmonary MRI is the elimination of the high radiation exposure of lung CT. One CT is equal to to 100–200 chest radiographs. Depending on the age of the patient, the diagnostic-induced triggering of a malignant tumor occurs statistically after approximately 1,000 thoracic CT examinations. Pediatric pulmonologists rarely ignore this argument. About 80–90% of all CT examinations of the lung could be replaced by a lung MRI examination with good diagnostic validity — probably even more in the future. A second point is the fact that, in certain diseases, the lung MRI is not only of equal value to CT, but in individual cases MRI can even be superior. Additional information — such as the detection of pus in pneumonia with abscess formation [7] or signal-reduced lymph nodes in sarcoidosis [8] — makes MR diagnostics interesting for pediatric pulmonologists.

## Indications

It is essential to identify the so-called MR-plus pathologies together with the referring partner. MR-plus pathologies are diseases that are particularly well described on MRI because many protons accumulate in the pathological lung process [9]. These include inflammation-caused accumulation of fluids in the alveolar space as well as all infiltrative processes, tissue proliferation and lung metastases.

This implies, according to current knowledge, that so-called MR-minus pathologies are not to be examined primarily with MRI, but preferentially by CT. These include diseases with a loss of protons, such as emphysema, little cysts and fibrosis at an early stage. According to current knowledge, the search for metastases in osteosarcoma also belongs in CT because these metastases typically calcify at an early stage and thus elude MRI diagnostics.

Although cysts and pulmonary fibrosis are generally counted as MR-minus pathology, discrete findings can now also be made using increasingly better MR examination techniques or special techniques. One should be aware, however, that the presentation and interpretation of such discrete findings on MRI is not as easy as with the changes usually found in MR-plus pathology. This means that the boundaries have become blurred: “In-between pathologies” could be called diseases that, depending on the referrer’s familiarity and the radiologist’s expertise, can be diagnosed at MRI too. These include lung cysts and congenital pulmonary airway malformation (CPAM) Types 1 and 2 [10].

## Quality criteria

A good MRI examination has the following criteria as minimum requirements:

- The central vessels should be sharply definable at least up to the 4th-order branching.
- The bronchi should be clearly recognizable by a bronchial wall at least up to the 2nd-order branching.
- The in-plane resolution should be a maximum of 1×1 mm. The slice thickness should not be more than 4 mm at 1.5-tesla (T) and not more than 3 mm at 3.0-T MRI devices.
- Movement artifacts caused by the heart and thoracic wall should not be detectable or should be minimal enough not to interfere with assessment.
- There should be no dorsal atelectases (in sedated children) affecting the assessment.

In accordance with these quality criteria, the radiologist — regardless of the diagnostic assessment — must first formally evaluate the lung MRI examination according to a three-category scheme: Category A, the examination was technically successful without any relevant movement artifacts and the question can be answered; Category B, the examination shows movement artifacts but allows for a sufficiently reliable answer to the clinical question; or Category C, the examination shows movement artifacts or dorsal dystelectases from sedation such that a diagnostic evaluation is not possible.

**Fig. 1:**
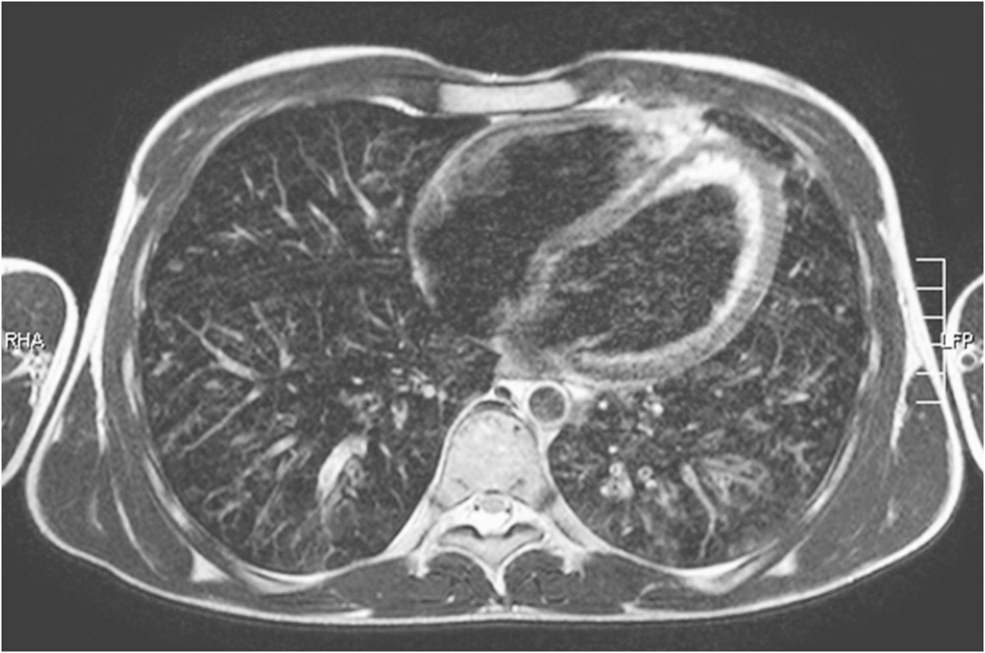
Normal, good-quality lung MR in a 9-year-old girl (axial T2-weighted turbo spin-echo respiratory-triggered examination, parameters in Table 1). The arteries and bronchi are of equal width and have a dark lumen as well as a delicate signal-rich wall. The veins are predominantly bright, their walls indistinct. There is no relevant cardiac or breathing artifact

Only Categories A and B examinations should be evaluated radiologically (Fig. 1; Table 1). In contrast, Category C examinations have to be formally classified as diagnostically inadequate and a timely CT examination should be organized by the pediatric radiologist, preferably on the same day. It should not be the task of the clinical colleague to commission the CT examination in the case of a non-diagnostic lung MRI. At the end of the day the clinician needs an image-based diagnosis, regardless of the method. This formalized approach will earn you the trust of your clinical colleagues.

## Technique

Disagreement on the optimal sequences for a diagnostically sufficient depiction of pathological lung findings is regarded as one of the causes for the still-low prevalence of lung MRI examinations. It is also evidence that there is not *the* optimal sequence. Sequences that are adapted to different and essential questions have been published in various reviews and consensus papers (Table 1) [11].

**Table 1:** Magnetic resonance imaging sequences by clinical indication

Clinical question	Sequences	Respiratory compensation
Tumors, pneumonia, airways		
	T1-weighted 3-D gradient echo T1-weighted 2-D gradient echo	Breath-hold
	T2-weighted fast spin echo	Multiple breath-hold
	T2-weighted turbo spin echo with rotating phase coding	Multiple breath-hold
	T2-weighted turbo spin echo	Respiratory triggering
	T2-weighted turbo spin echo with fat suppression	Respiratory triggering or multiple breath-hold
	Diffusion-weighted imaging	Respiratory triggering or multiple breath-hold
Lymph nodes and bone metastases		
	T2-weighted short tau inversion recovery	Respiratory triggering
Vascular imaging, perfusion		
	T1-weighted gradient echo	Breath-hold
	T1-weighted 3-D gradient echo (echo sharing)	Breath-hold
	T1-weighted/T2-weighted steady-state gradient echo	Breath-hold

In addition, there are several very detailed compilations with suitable lung MRI sequences, of which the publication of Ohno et al. [12] on nodule detection and the publication of Baez et al. [13] on pediatric radiology list the MR parameters in detail. However, the problem with such compilations is that the different sequences are presented in detail regarding their parameters. However, with this variety of sequences, it remains open which of the sequences mentioned should then be used in the daily routine to provide the best diagnostic results. Therefore, next we describe some basic considerations about sequence selection that might be helpful. The focus is on the evaluation of lung parenchyma and the examination of small children up to 8 years of age.

## General recommendations for good lung magnetic resonance imaging examinations in young children

### Breath-holding sequences are naturally not suitable for young children.

This also applies to older children with limited lung function [14]. Therefore only sequences that are acquired in expiration with different trigger techniques [15] or that have recently been offered as “self-gated sequences” can be considered [16].

### Pulmonary pathologies have so far been examinated with a strong T2-contrast.

Today, most pediatric radiology institutions exclusively use T2-weighted breath-triggered turbo spin-echo (TSE) sequences with long echo traction; these sequences show good T2-contrast of the pathologies against the dark lung. This results in largely artifact-free, sharp images. Today, these sequences are the workhorse of lung MRI. They can also be performed with fat saturation, which increases the T2 weighting [6].

### T1-weighted ultrashort echo time (UTE) sequences have long been discussed as an option in lung imaging.

These gradient echo sequences are theoretically well suited for the representation of pathological processes in the lung [17]. Infiltrations and tissue structures are shown isointense to soft tissue. The image impression is comparable to that of a lung CT without contrast medium [18]. Until now, it was not technically possible to read the signal immediately after excitation. The reading of the signal was delayed, which led to an extreme signal loss from susceptibility. Only in the last 2–3 years has it been possible to technically optimize the MRI devices in such a way that echo times in the microsecond range are possible (Table 2). This means that the old idea of the UTE sequence can now be put into practice [19]. This technology could revolutionize lung imaging in MRI [20].

**Table 2:** Simple lung base protocol in infants and young children at 3.0 tesla

Parameter	T2 turbo spin echo, respiratory triggered	T2 turbo spin echo with fat suppression, respiratory triggered	T1-W 3-D ultrashort echo time sequence, respiratory triggered
Repetition time (ms)	1,000–2,500^a^	1,000–2,500^a^	4.1
Echo time (ms)	56	57	0.07
Fip angle	140°	140°	6°
Number signals acquired	1	1	1
Section orientation	Axial and coronal	Axial	Coronal + reconstruction
Field of view (mm)	340	340	340
Phase resolution	70%	70%	100%
Matrix	320×168	320×168	360×360
Voxel size (mm)	1.1×1.5×3.0	1.1×1.5×3.0	0.86×0.86×0.86
PAT (parallel acquisition techniques) mode	PAT 2	PAT 2	Not applicable
Contrast medium	Not applicable	Not applicable	No
Motion compensation	Respiratory triggering	Respiratory triggering	Self-gated
Acquisition time	2–6 min^a^	2–6 min^a^	6–8 min^a^

^a^ Depending on the child’s respiratory rate

### Should breath-held sequences be used in older children?

We recommend the stable breath-triggered T2-W TSE sequences for older children. However, if breath-held sequences are to be used for time reasons, single-shot sequences with T2 contrast (half-Fourier single-shot turbo spin-echo sequences, or HASTE) or mixed T1/T2 contrast sequences (true fast imaging with steady-state free precession sequences, TRUFI) have unfortunately not proved successful in clinical routine. Several studies have shown that pathological findings (both in the alveolar space and in the pulmonary interstitium) are more difficult to detect with these two sequences than in the breath-triggered T2-W TSE sequences. However, if a T2-weighted sequence is to be performed using the breath-holding technique, a so-called T2-W TSE multi-breath-hold sequence in older children is considered superior to T1/T2 TRUFI single-shot sequences and T2-W HASTE [21, 22].

T1-W UTE sequences can also be executed as 2-D architecture in a short respiratory arrest. They then no longer last 6–8 min — like the self-gated T1-W 3-D UTE sequences with central k-space sampling — but rather show a very short acquisition time of only a few seconds (13 s) using breath-holding technology. However, the slices are 2–3 mm wide and a reconstruction of other orientations is therefore only possible to a limited extent. It should be noted that breathing sequences always convey a slightly different image impression because of the inspiratory position than the breath-triggered images taken in expiration.

### Do you need more sequences than T2-weighted lung images or the new T1-W UTE sequences?

No, additional sequences are an absolute exception in MR diagnostics of the lung. The basic rule should be: Keep it simple. Even in the case of lung tumors, the administration of a contrast agent does not seem to be necessary, although no systematic studies have been carried out in children. Only in the case of abscessing pneumonia has the use of a breath-triggered T1-W gradient echo sequence proved to be effective because abscesses in T2-W sequences can have the same signal intensity as the infiltrated lung tissue. In these rare cases, a diffusion-weighted imaging (DWI) sequence can help to differentiate between serous and purulent fluid accumulations (Table 3).

**Table 3:** Additional sequences as required in infants and young children at 3 tesla

Parameter	T1-W 2-D gradient echo, respiratory triggered	Diffusion-weighted imaging, respiratory triggered	Time-resolved angiography
Repetition time (ms)	120	1,500–1,700^a^	2.8
Echo time (ms)	2.5	73	1.06
Flip angle	70°	NA	16°
Number signals acquired	1	5	10
Section orientation	Axial	Axial	Coronal
Field of view (mm)	340	320	250
Phase resolution	70%	80%	50%
Matrix	384x202	192x115	256x128
Voxel size (mm)	0.9x1.3x3.0	2.1x1.7x3.0	1.0x1.0x1.3
PAT (parallel acquisition techniques) mode	PAT 2	PAT 2	PAT 2
Contrast medium	Yes	No	Yes
Motion compensation	Respiratory triggering	Respiratory triggering	-
Acquisition time	2–6 min^a^	2–6 min^a^	3 s per frame

^a^ Depending on the child’s respiratory rate

*min* minutes, *NA* not applicable, *s* seconds

**Fig. 2:**
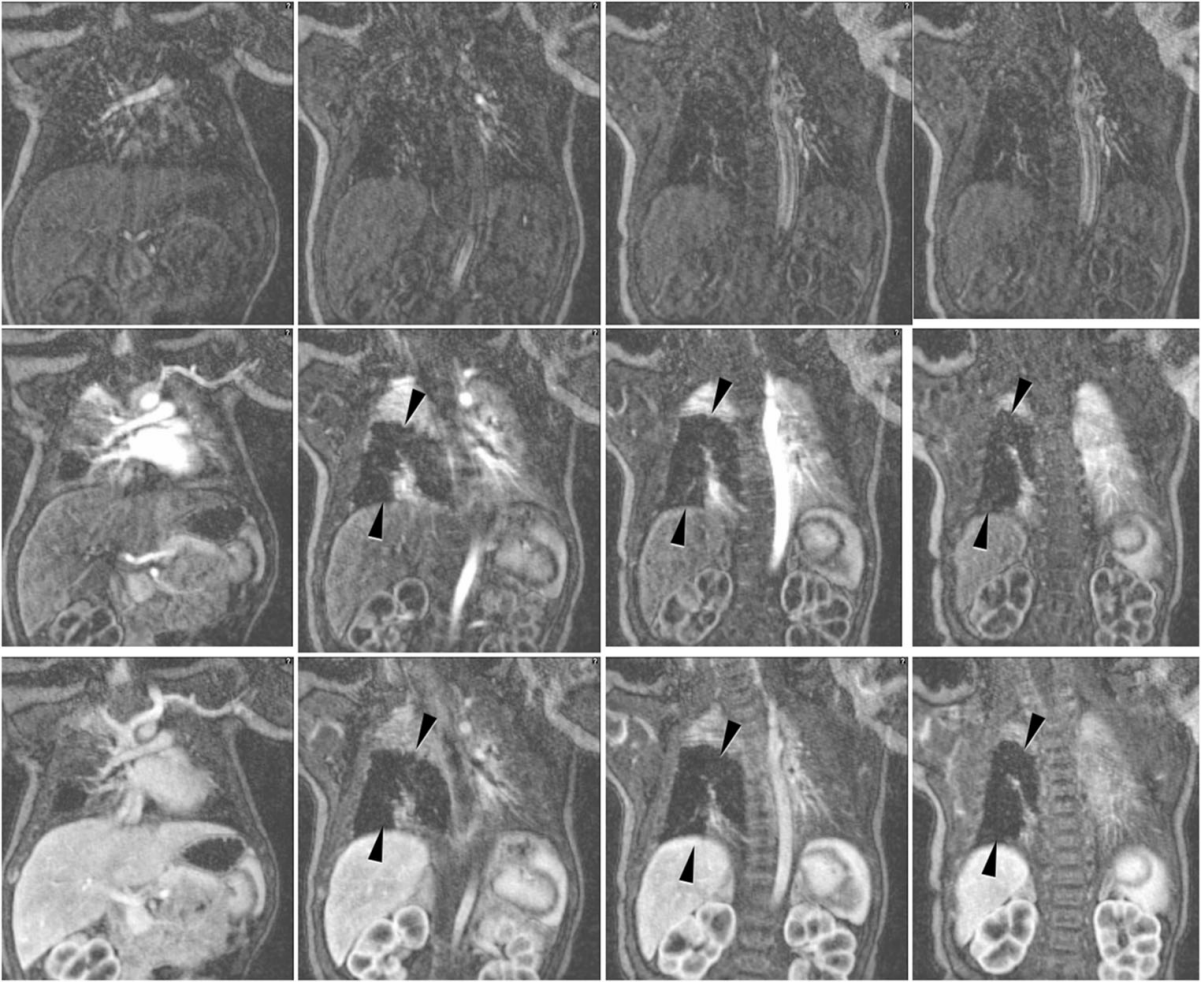
Coronal time-resolved lung MR angiography in a 4-day-old boy with congenital pulmonary airway malformation of the right lung (MR parameters in Table 2). With a temporal resolution of 2–3 s, during the first pass of the contrast medium it is possible to distinguish the non-capillarized signal-free cystic lesion (*arrowheads*) from the normally contrast-enhancing lung tissue

Angiographic images of the thoracic blood vessels are only necessary for the characterization of rare vascular malformations and for the representation of the vascular situation in case of a suspected pulmonary sequestration. The corresponding sequence parameters are generally known and available. However, we use MR angiography in the form of fast dynamic temporally high-resolution MR angiographies of the thorax for a completely different aspect. With time-resolved angiography (time-resolved angiography with stochastic trajectories, or TWIST), the entire lung can be examined in 2–3 s. This makes it possible to identify the optimal contrast medium phase in the lung after the application of contrast medium in the first-pass procedure [23]. In the optimal contrast phase of the lung parenchyma, the non-contrasted areas of the lung can thus be well delimited. This makes it possible to detect cysts and bullae within the lung (Fig. 2). Local emphysema can also be well visualized with this first-pass technique.

### Lung MRI can be performed on both 1.5-T MR devices and 3.0-T MR devices.

On 1.5-T devices, the vascular architecture of the lung is shown somewhat better than at 3.0 T. But we did some comparative studies on the same child: the pathology showed excellent visibility at 3.0 T against the slightly dark lung (e.g., in case of small metastases). Therefore we now only perform lung MRI at 3.0 T. However, this is our personal preference and I do not see a reason to prefer 3.0-T devices over 1.5-T devices. Both field strengths are well suited for lung imaging.

## Plus-pathology of the lung at magnetic resonance imaging

These diseases typically show findings that are easy to diagnose on MRI. Assuming an adequate examination technique, MRI diagnostics is on a par with CT for these diseases. This includes all lung diseases with alveolar exudation and infiltration by infections [24, 25], tissue proliferation such as tumors or metastases [22], malformations such as sequesters [26] and of course cystic fibrosis, which is particularly important in pediatric pulmonology [27].

### Inflammatory changes

**Fig. 3:**
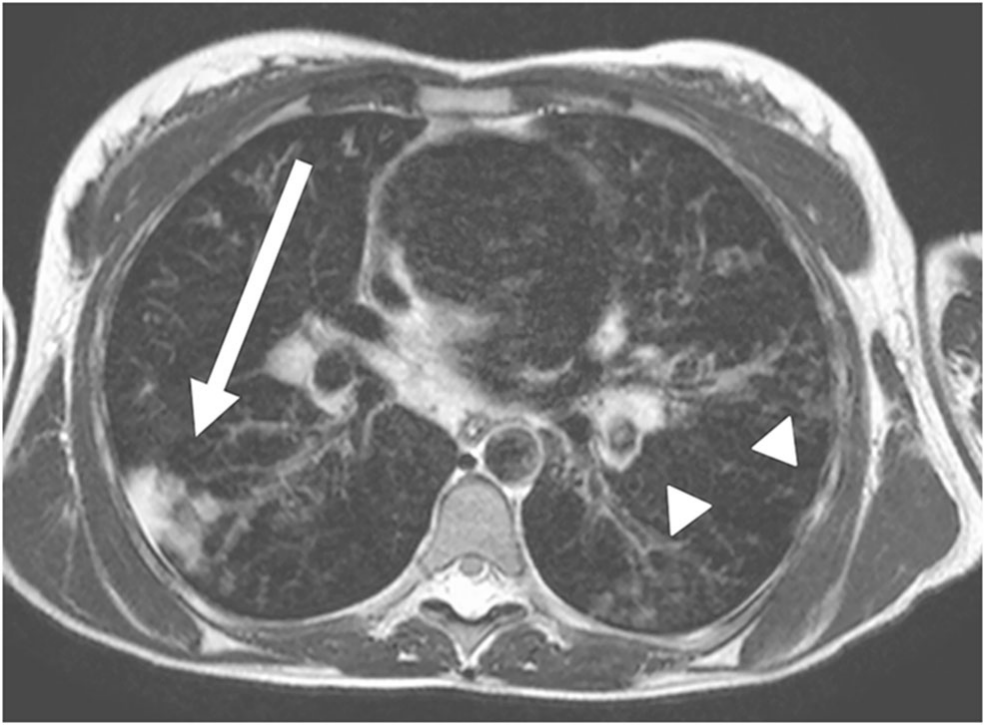
Bronchopneumonia on lung MR in a 10-year-old girl (axial T2-weighted turbo spin echo, parameters in Table 2). There is alveolar exudation and infiltration in bronchopneumonia in the right lower lobe (*arrow*) and, to a lesser extent, in the left lower lobe (*arrowheads*)

**Fig. 4:**
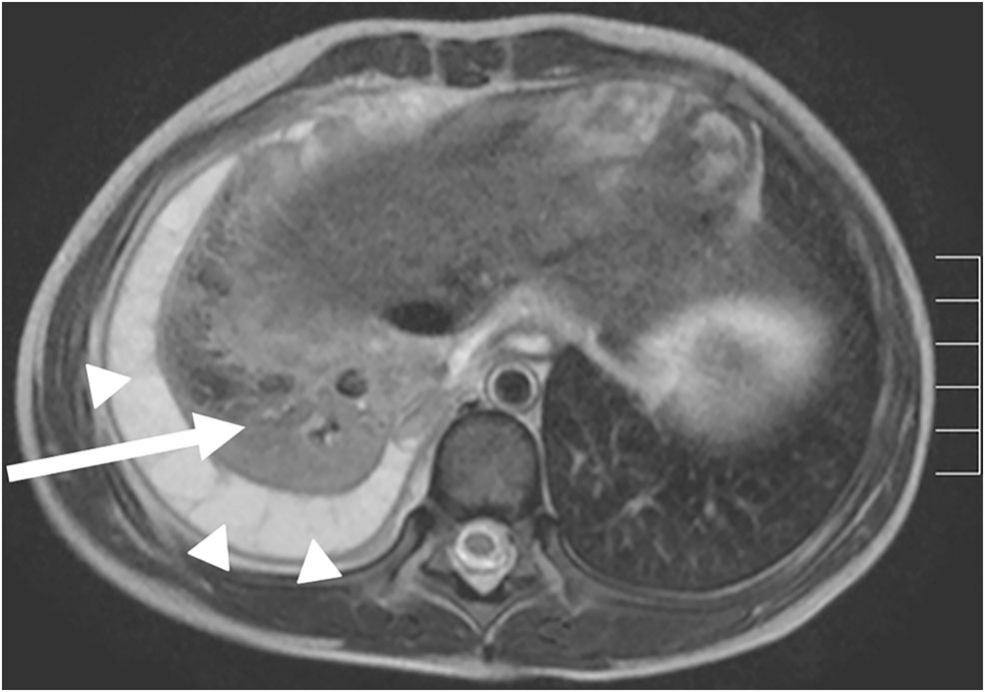
Pneumonia on lung MR in a 5-year-old girl (axial T2-weighted turbo spin echo, parameters in Table 2). There is almost complete alveolar infiltration in the right lower lobe (*arrow*) caused by lobar pneumonia. Note pleural effusion with fibrinous septation (*arrowheads*)

**Fig. 5:**
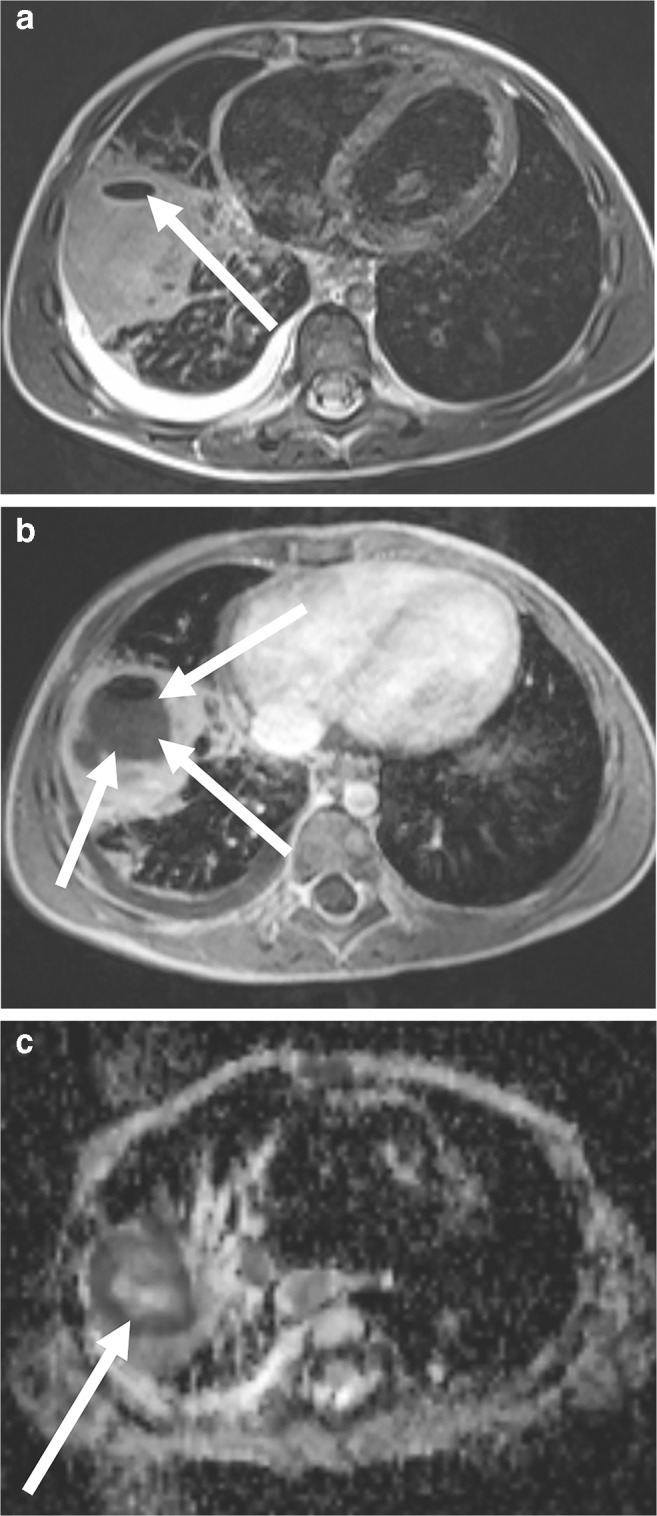
Lobar pneumonia with an abscess on lung MR in a 3-year-old girl (MR parameters in Tables 2 and 3). **a** On axial T2-weighted turbo spin-echo image, the abscess is not clearly distinguishable from the surrounding inflammatory tissue. It only demarcates itself by ventral air accumulation (*arrow*). **b** In axial T1-W gradient echo sequence after contrast medium administration, the abscess (*arrows*) is clearly distinguishable from the inflammatory area. Note that the administration of a contrast agent for lung examinations is almost only necessary for this indication. **c** Apparent diffusion coefficient (ADC) map from the axial diffusion-weighted imaging sequence: The low ADC values (*arrow*) prove the purulent content within the cave

**Fig. 6:**
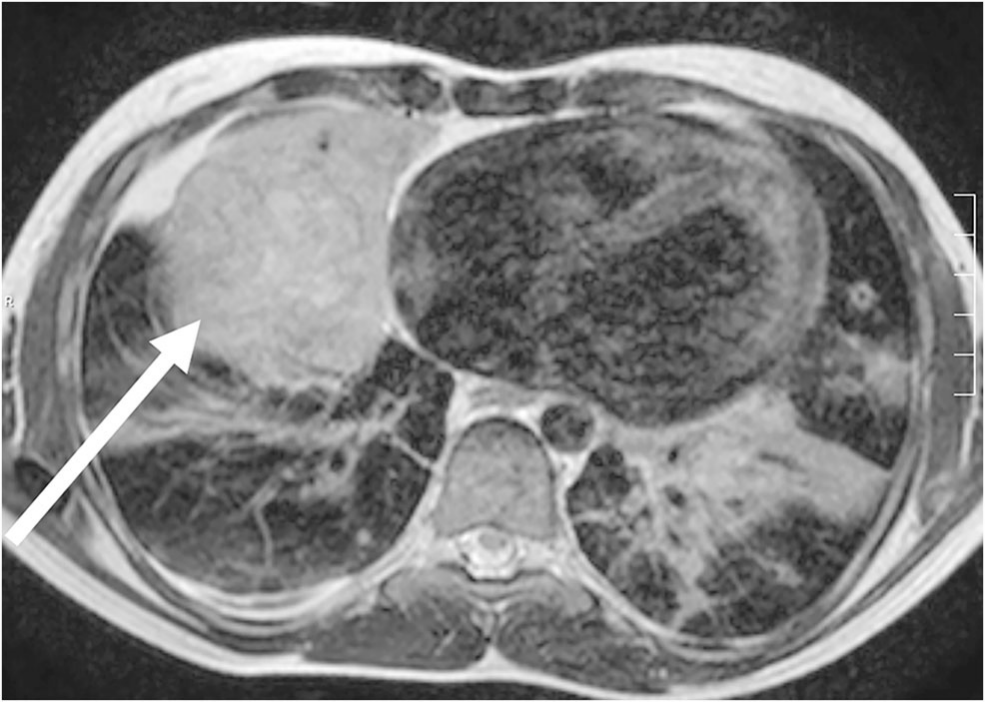
Lung MR in a 16-year-old girl with severe combined immunodeficiency. Axial T2-weighted turbo spin-echo image (MR parameters, Table 2) shows an inflammatory myofibroblastic tumor (*arrow*) in the right middle lobe

**Fig. 7:**
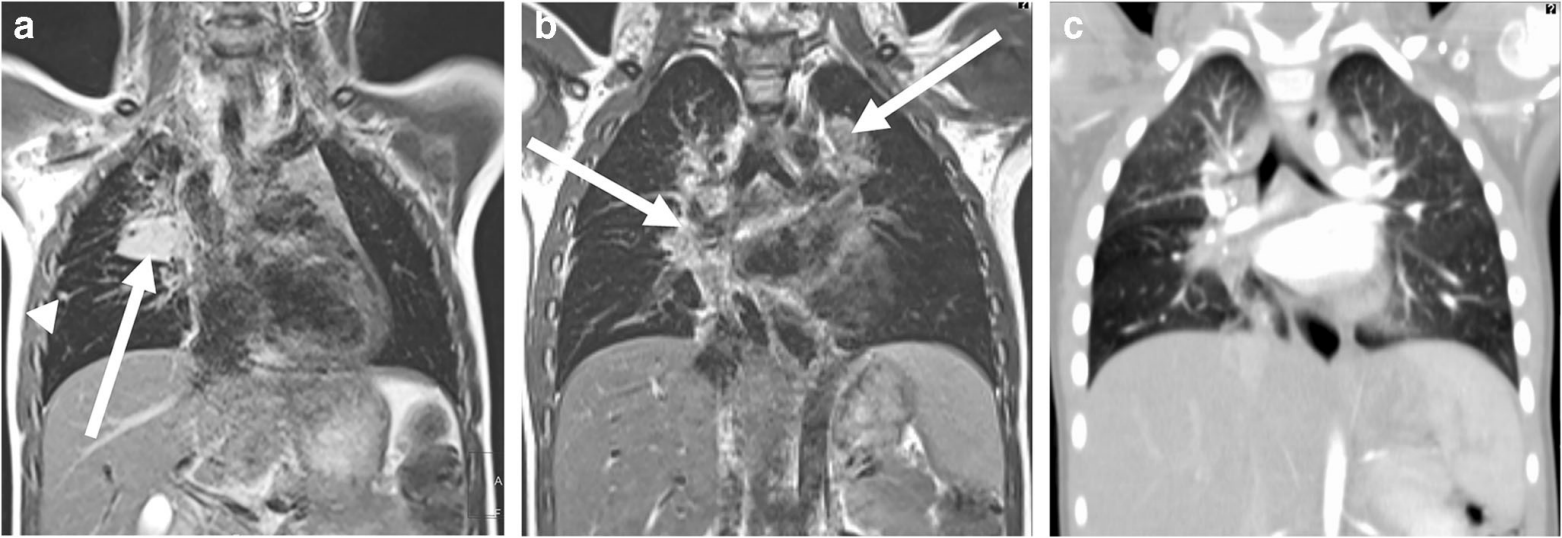
MRI of lung tuberculosis in a 1-year-old boy (MR parameters, Table 2). **a** Coronal T2-weighted turbo spin-echo image shows an infiltrate (*arrow*) in the right upper lobe. Note also the 3-mm nodular infiltrates (*arrowhead*) in the right lung. **b** Coronal T2-weighted turbo spin-echo image shows swelling of hilar lymph nodes (*arrows*) and evidence of central peribronchial infiltrations. **c** Coronal reformat of a low-dose contrast-enhanced CT for comparison

**Fig. 8:**
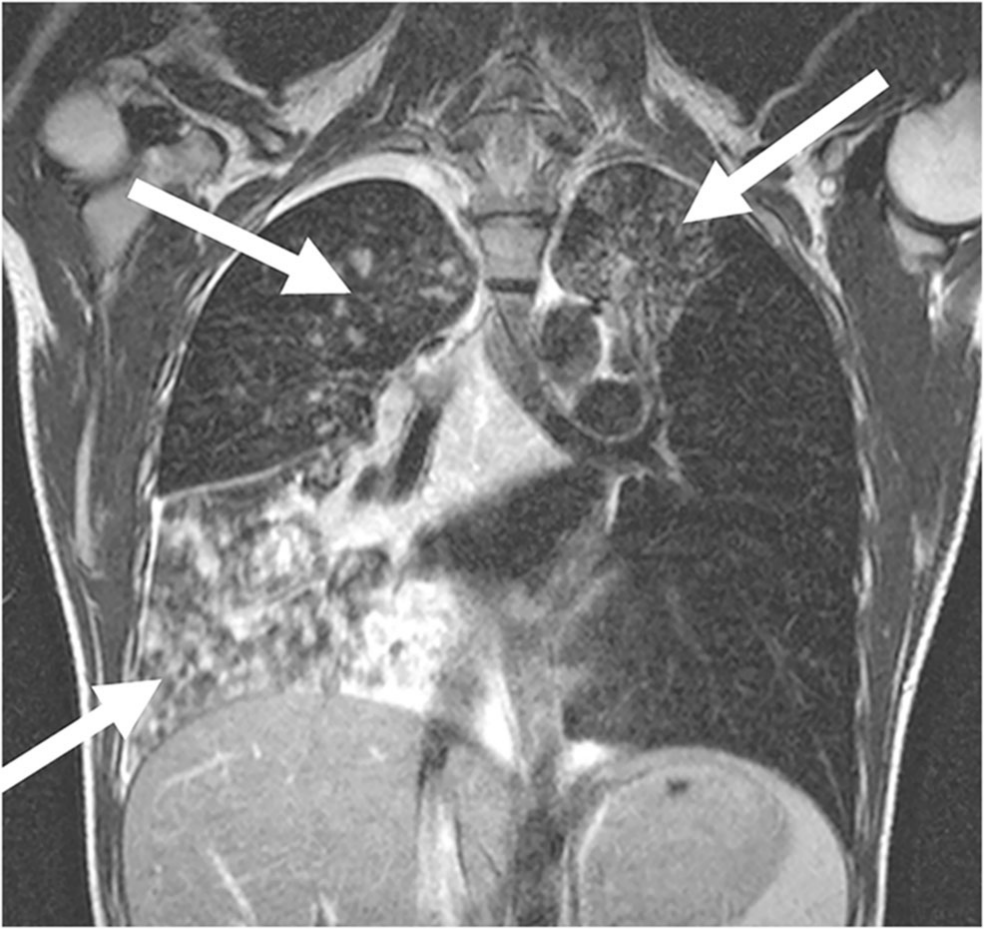
MRI of tuberculosis in a 12-year-old girl. Coronal T2-weighted turbo spin-echo image (MR parameters in Table 2) demonstrates that in older children, the tuberculous infiltrates (*arrows*) are often multi-focal and look similar to bronchopneumonia

Abnormality caused by inflammation is characterized by accumulation of fluid in the alveolar space and therefore shows high MRI signal (Figs. 3 and 4) [24, 25]. MRI is not commonly used in the examination of simple pulmonary inflammation, but it can be useful if complications, for example abscess formation, are suspected (Figs. 5 and 6) [7]. In lung tuberculosis, the characteristically enlarged lymph nodes and additional abnormalities can be visualized by MRI (Figs. 7 and 8).

### Tumorous lesions

**Fig. 9:**
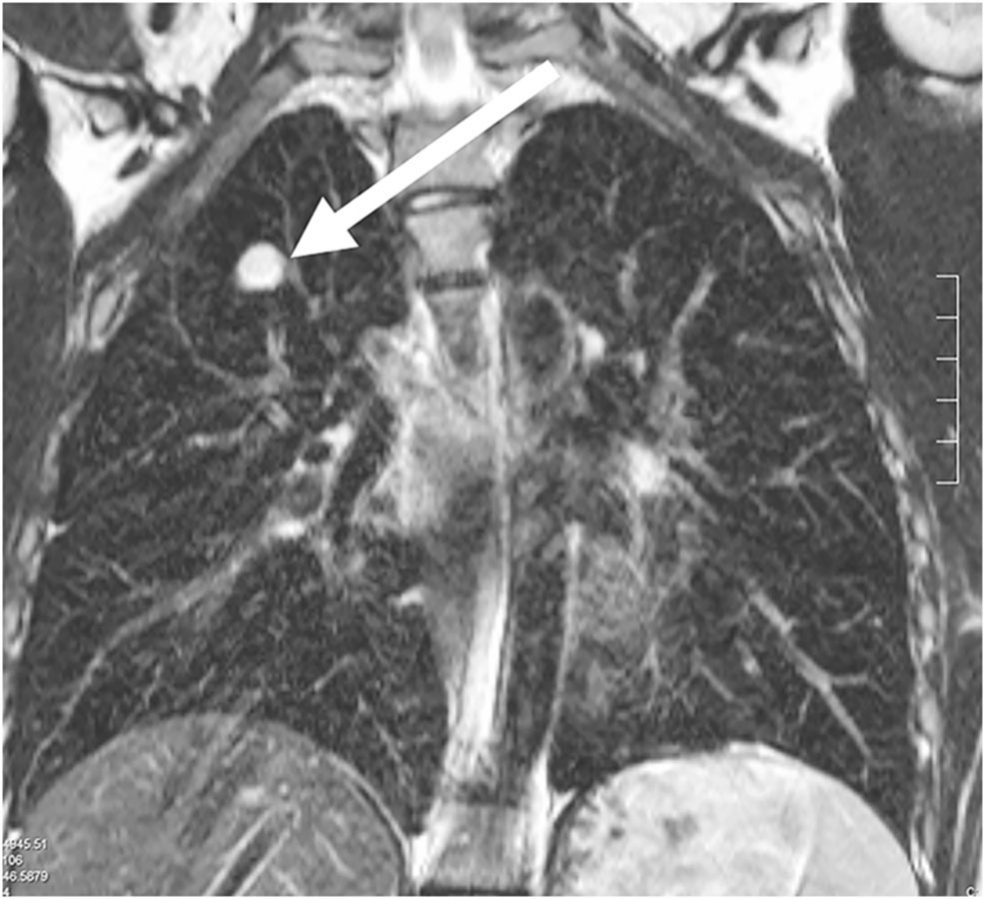
Hamartoma on lung MR in a 16-year-old girl (coronal T2-weighted-turbo spin echo, MR parameters in Table 2). Image shows a hamartoma (*arrow*) in the right upper lobe. The lesion was an incidental finding on a chest radiograph taken because of a cough. On MR, the nodule presents homogeneous hyperintensity

**Fig. 10:**
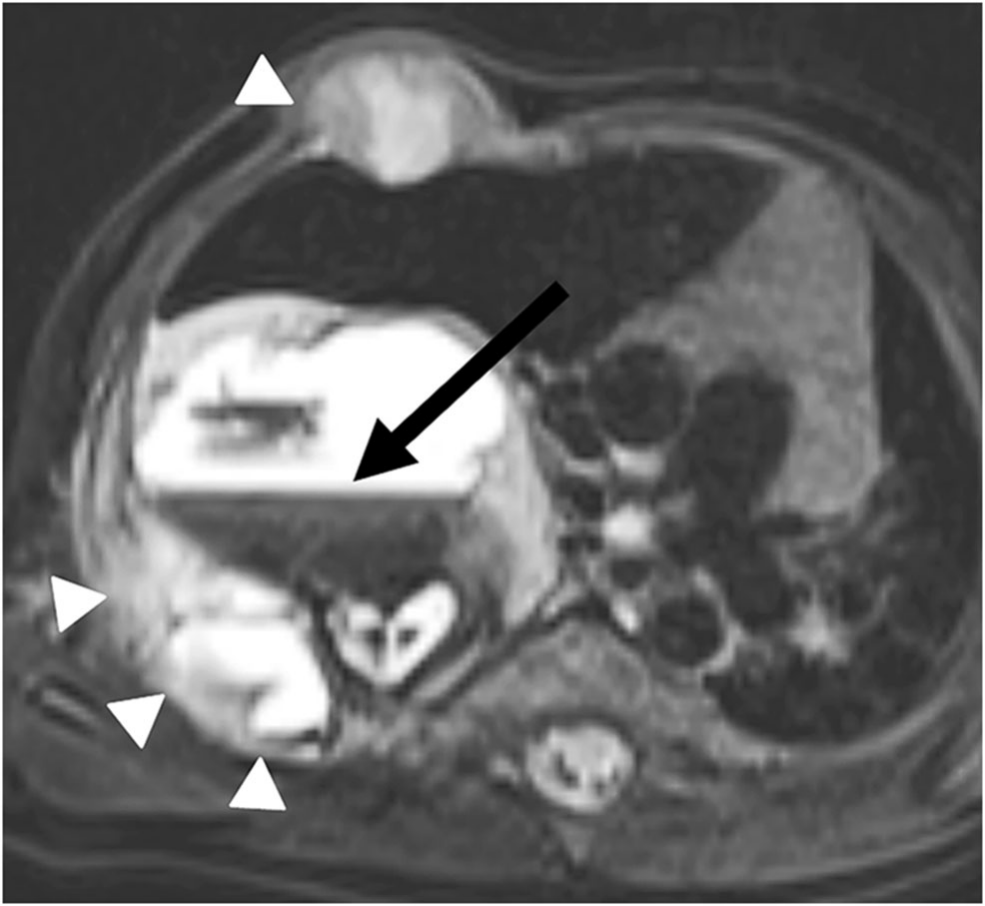
Multifocal mesenchymal hamartoma on lung MR in a 1-year-old boy. Axial fat-suppressed T2-weighted-turbo spin-echo image (MR parameters in Table 2) shows a multifocal mesenchymal hamartoma of the right thoracic wall with a large intrapulmonary portion in a newborn. The tumor originates primarily from the ribs (*arrowheads*), but manifests intrapulmonally. Blood-liquid levels (*arrow*) are a typical sign of associated aneurysmal bone cysts

**Fig. 11:**
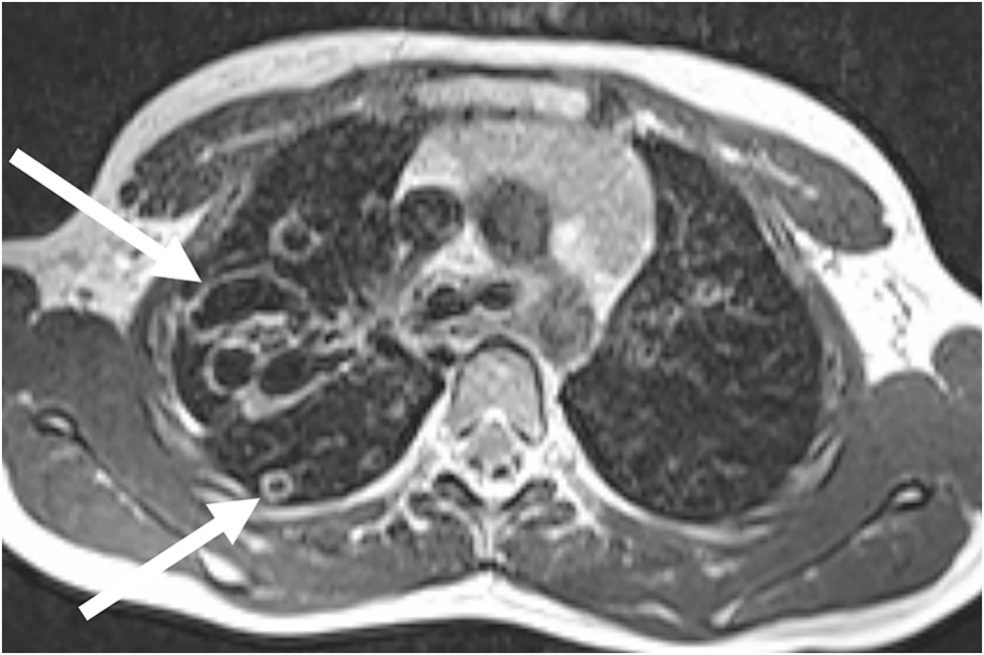
Pulmonary papillomatosis on lung MR in a 5-year-old girl. Axial T2-weighted turbo spin-echo image (MR parameters in Table 2) shows the typical manifestation of pulmonary papillomatosis, with round nodules that are solid or cystic and have a wall of varying thickness (*arrows*). They can grow to several centimeters and then lead to air-filled cavities, with significant destruction of the parenchyma

**Fig. 12:**
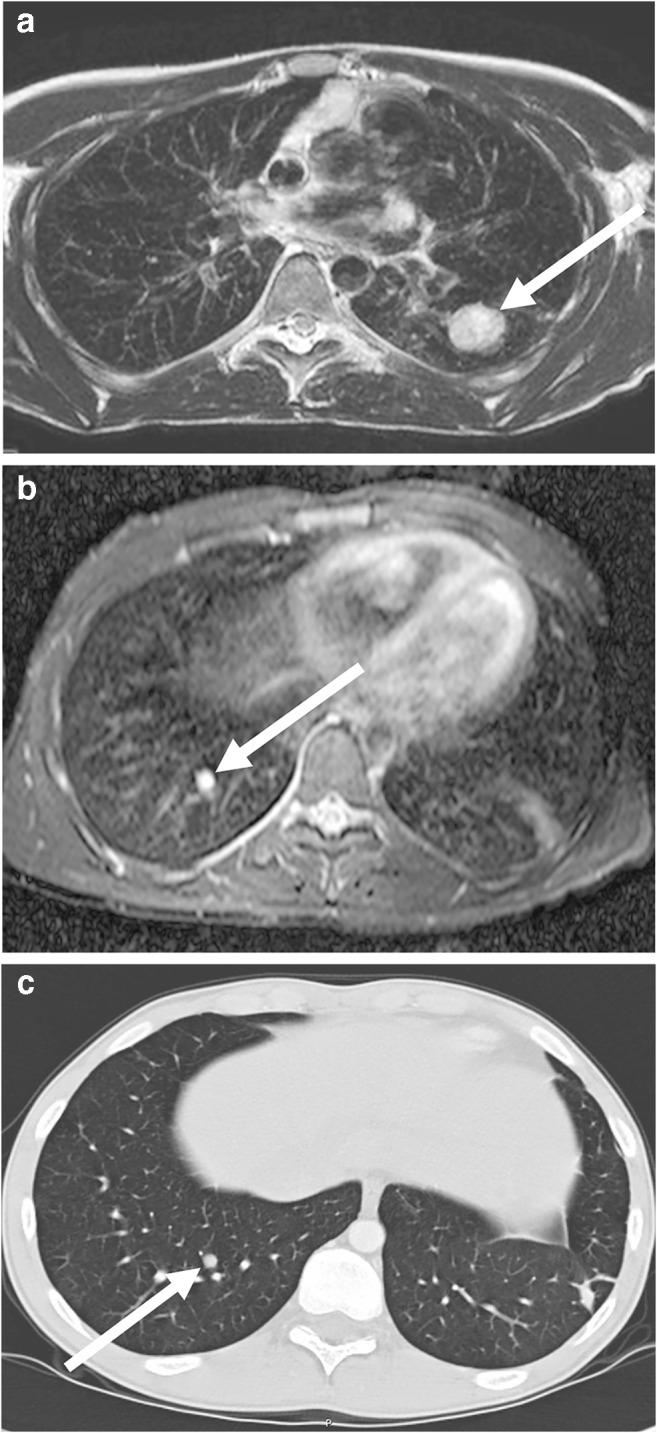
Axial lung MR in an 11-year-old boy with Ewing sarcoma and lung metastases (MR parameters in Table 2). **a** T2-weighted turbo spin-echo (TSE) image shows a 15-mm metastasis (*arrow*) in left upper lobe. **b** Fat-suppressed T2-weighted TSE image shows a 3-mm metastasis (*arrow*) in the right lower lobe. At MRI, metastases are just as easy to detect as they are at CT — they usually show a slightly brighter signal than that of vessels. This is especially true in heavily T2-weighted fat-suppressed sequences. **c** For comparison, axial CT image shows the same 3-mm metastasis (*arrow*) as the MR image in (**b**)

**Fig. 13:**
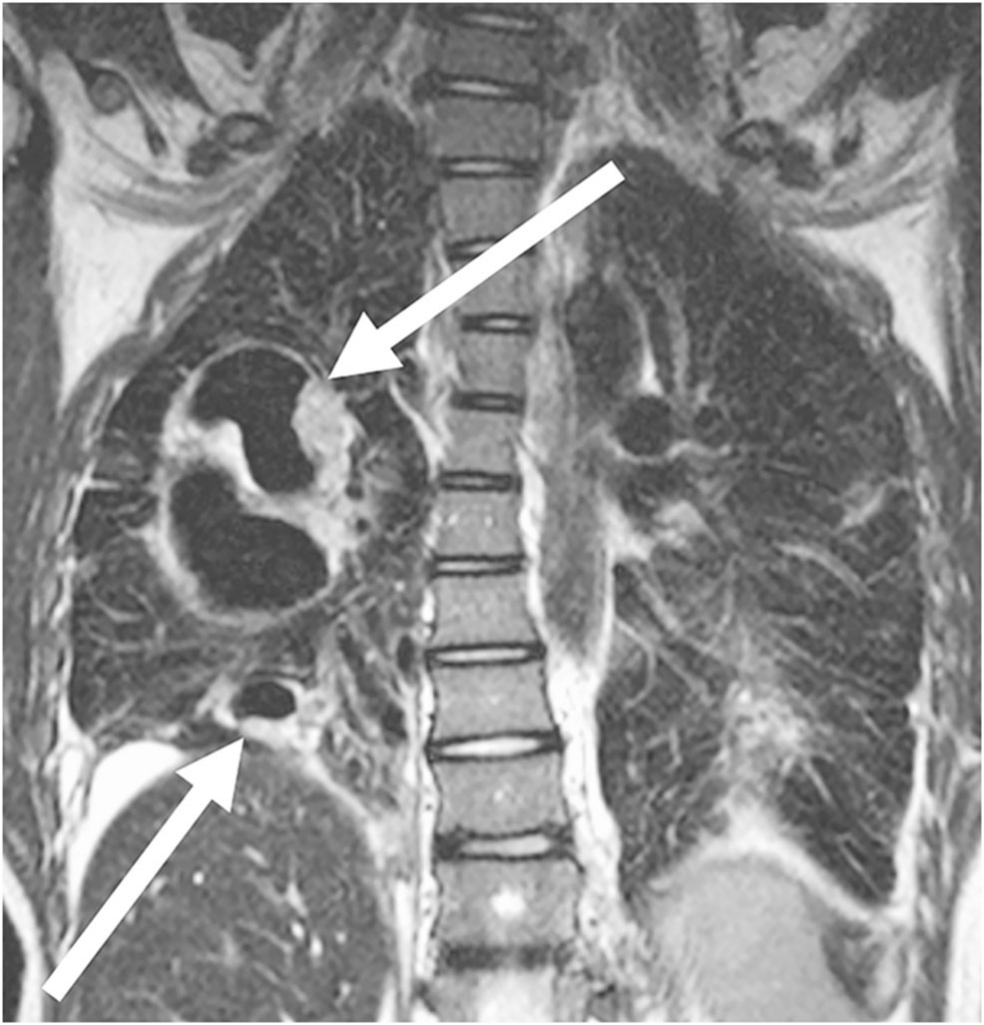
Rhabdomyosarcoma on lung MR in a 14-year-old girl. Coronal T2-weighted turbo spin-echo image (MR parameters in Table 2) shows partially cystic, partially necrotic metastases (*arrows*) from rhabdomyosarcoma

**Fig. 14:**
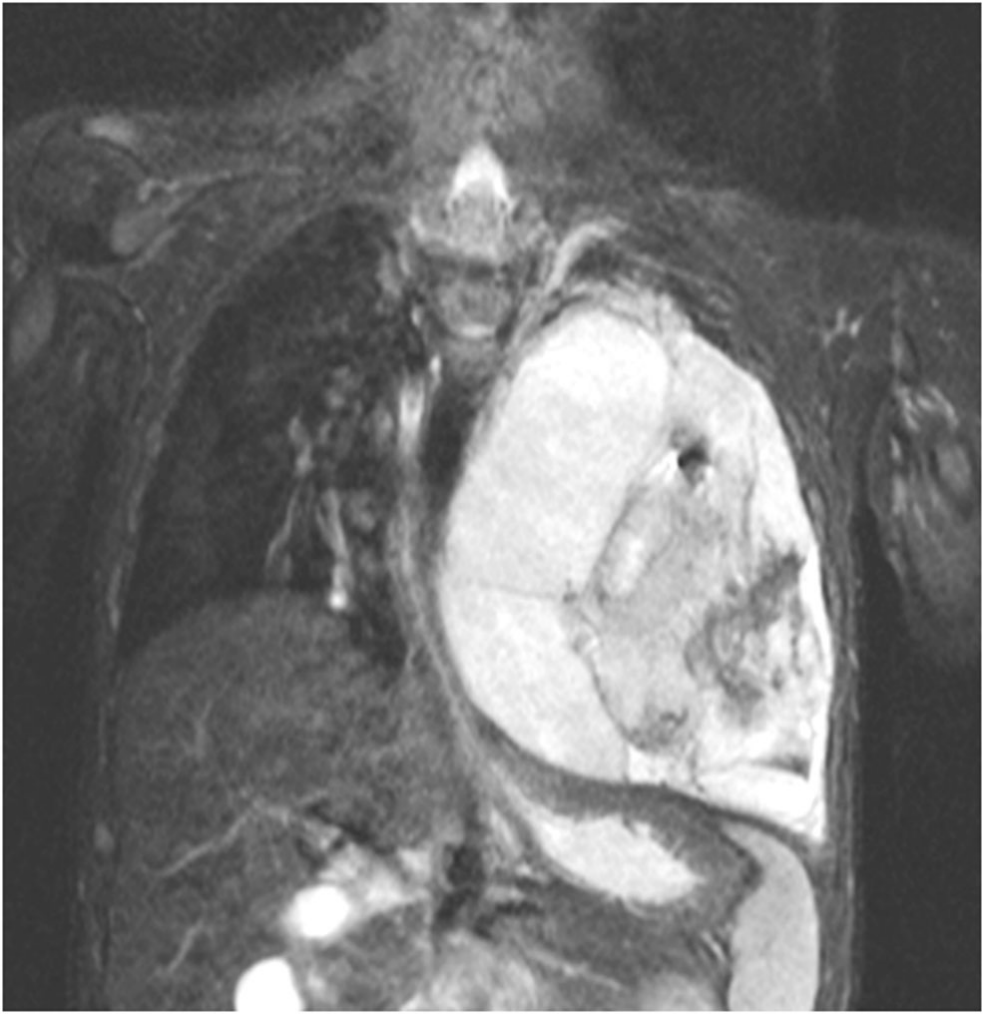
Lung MR in a 2.5-year-old boy with pleuropulmonary blastoma in DICER-1 syndrome. Coronal fat-suppressed T2-weighted turbo spin-echo image (MR parameters in Table 2) shows a partially cystic, partially solid tumor filling the entire left hemithorax

Tissue proliferation or tumorous lesions mostly show many protons in the pathological lung process and are therefore easy to detect on MRI (Figs. 9, 10, 11, 12, 13 and 14) [22]. There are some exceptions to this, e.g., largely calcified lesions like metastases of osteosarcoma. In some cases additional contrast-enhanced sequences can help to further characterize a lesion.

### Congenital malformations

**Fig. 15:**
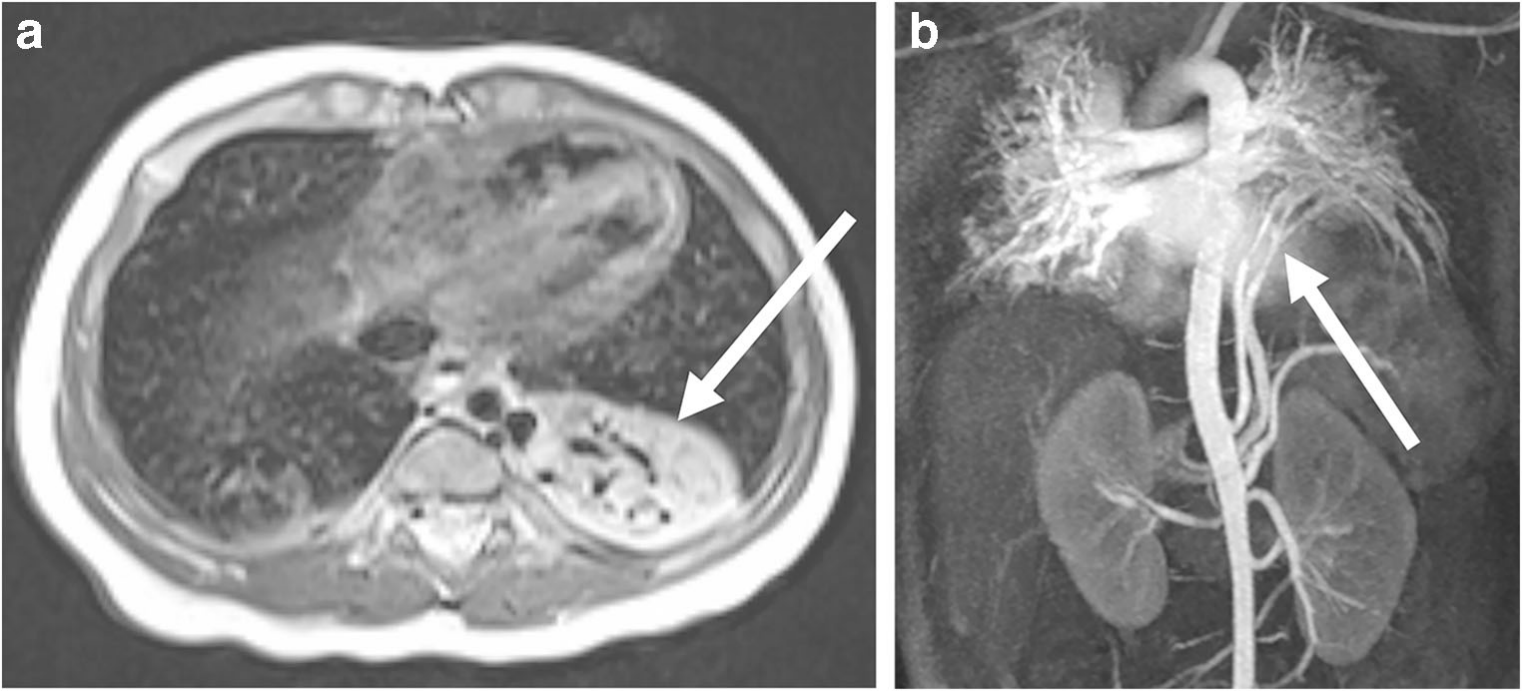
Pulmonary sequestration on lung MR in a 6-month-old boy. **a** Axial T2-weighted turbo spin-echo MR image (parameters in Table 2) identifies the supradiaphragmal lesion on the left (*arrow*) as a pulmonary sequestration by the low-signal vessels. **b** Coronal MR angiography proves the diagnosis by demonstrating the atypical arterial supply and venous drainage (*arrow*)

**Fig. 16:**
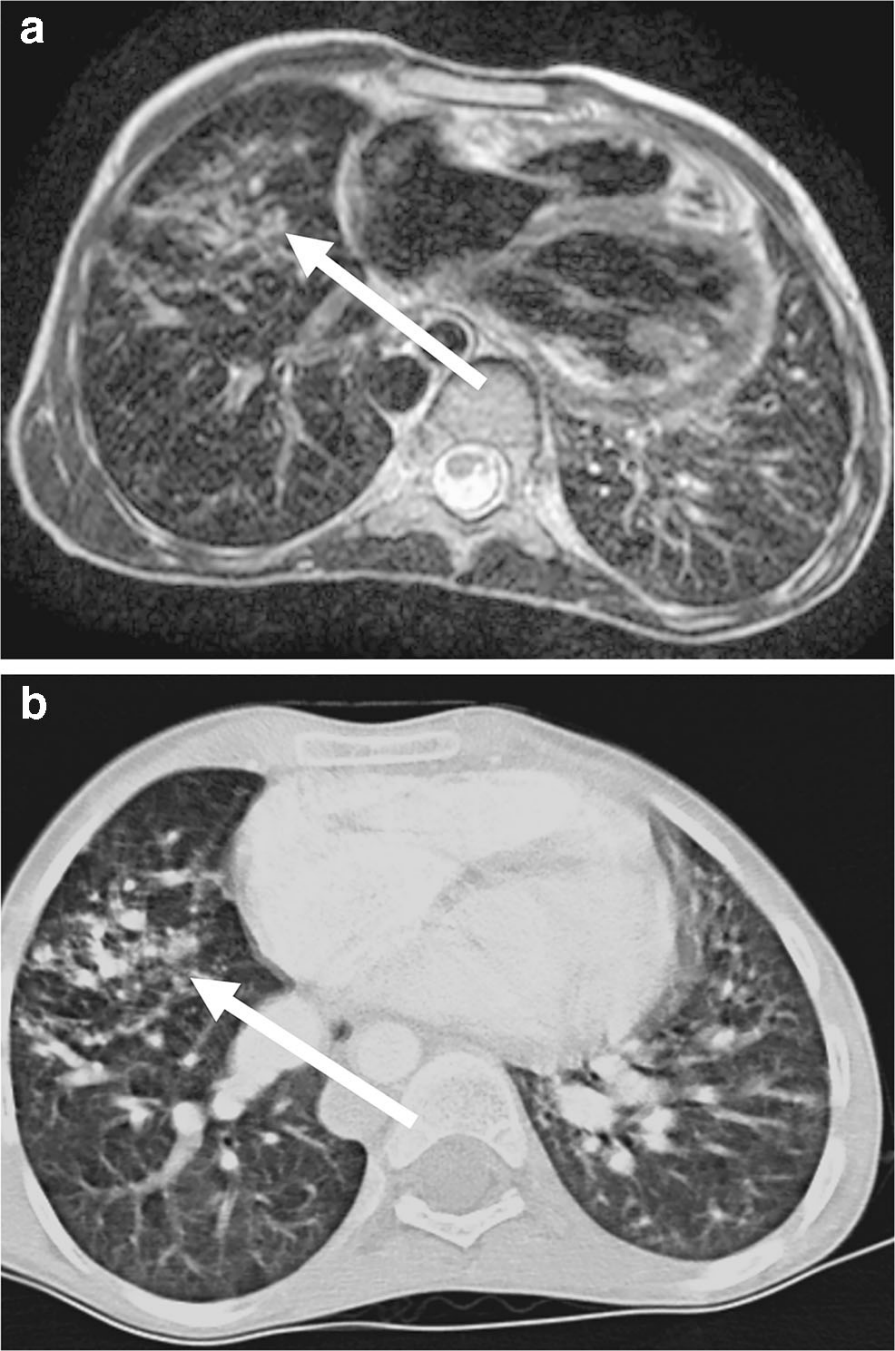
Pulmonary vascular malformation in a 9-year-old boy. **a** Axial T2-weighted turbo spin-echo MR (parameters in Table 2) shows multiple arteriovenous shunts (*arrow*). **b** Identical representation (*arrow*) in matching axial contrast-enhanced CT

Vascular and tissue malformations are naturally well detectable with MRI [26]. Pulmonary sequestration (Fig. 15) and vascular malformations (Fig. 16) are two of the few indications in which the administration of a contrast medium is indispensable to assess vascular anatomy.

**Fig. 17:**
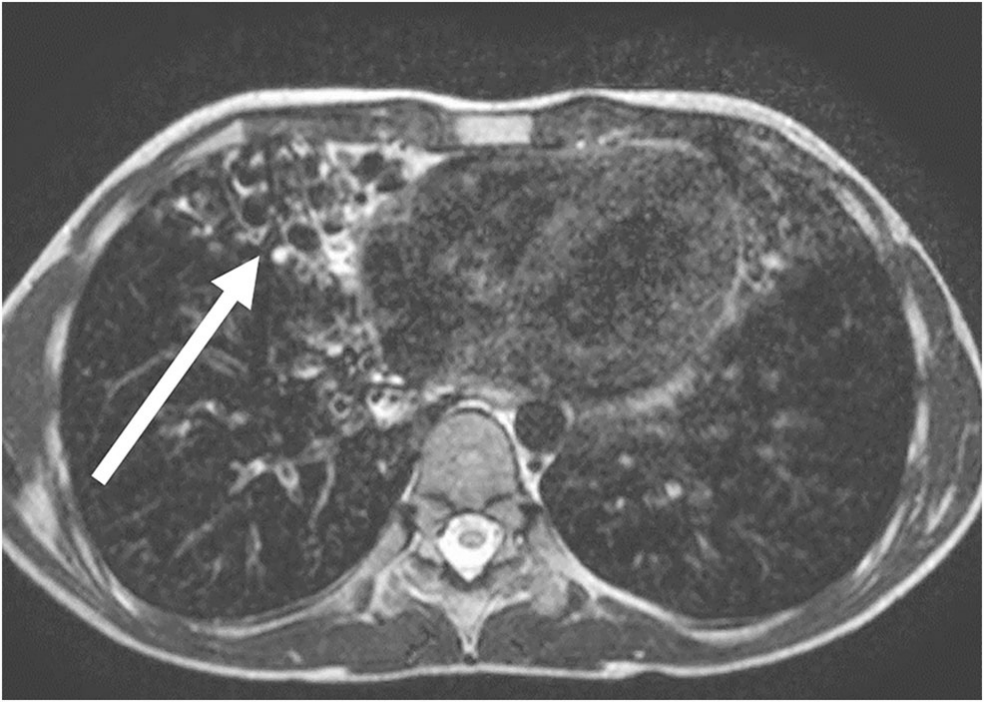
Lung MR in a 9-year-old girl with cystic fibrosis. Axial T2-weighted turbo spin-echo image (parameters in Table 2) shows multiple bronchiectases (*arrow*) in the right middle lobe. The bronchiectases are recognizable from the larger diameter and the thickened bronchial wall in contrast to the signal-free arteries

### Cystic fibrosis

**Fig. 18:**
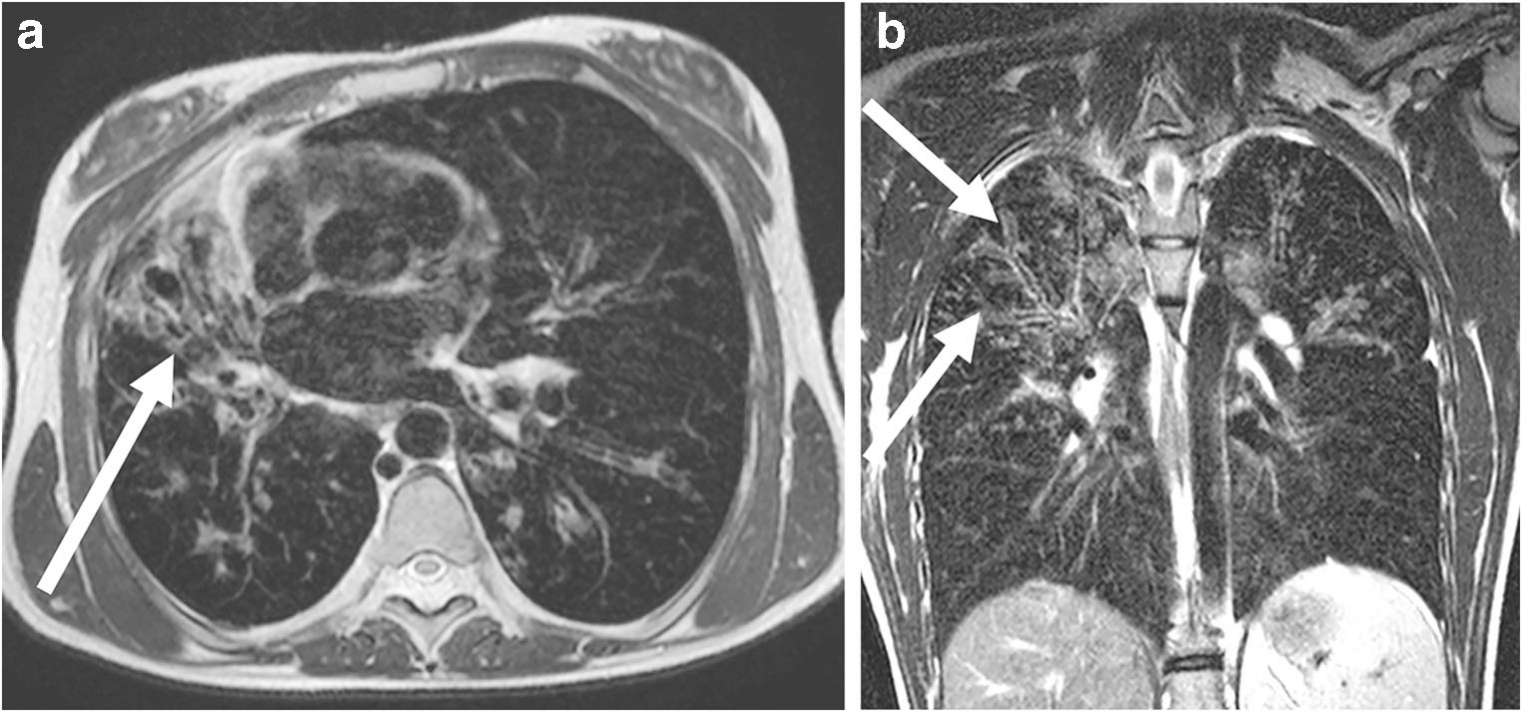
Lung MR in a 14-year-old girl with cystic fibrosis. **a, b** T2-weighted turbo spin-echo axial (**a**) and coronal (**b**) images show multiple bronchiectases among scarred strands, infiltrations and mucus plugging in the upper and middle lobes (*arrow* in **a**). Pulmonary scarring leads to thoracic asymmetry. Smaller pneumonic infiltrates (*arrows* in **b**) can be seen. Various scoring systems have been published and correlate well with the modified Bhalla CT score and the Chrispin–Norman score

**Fig. 19:**
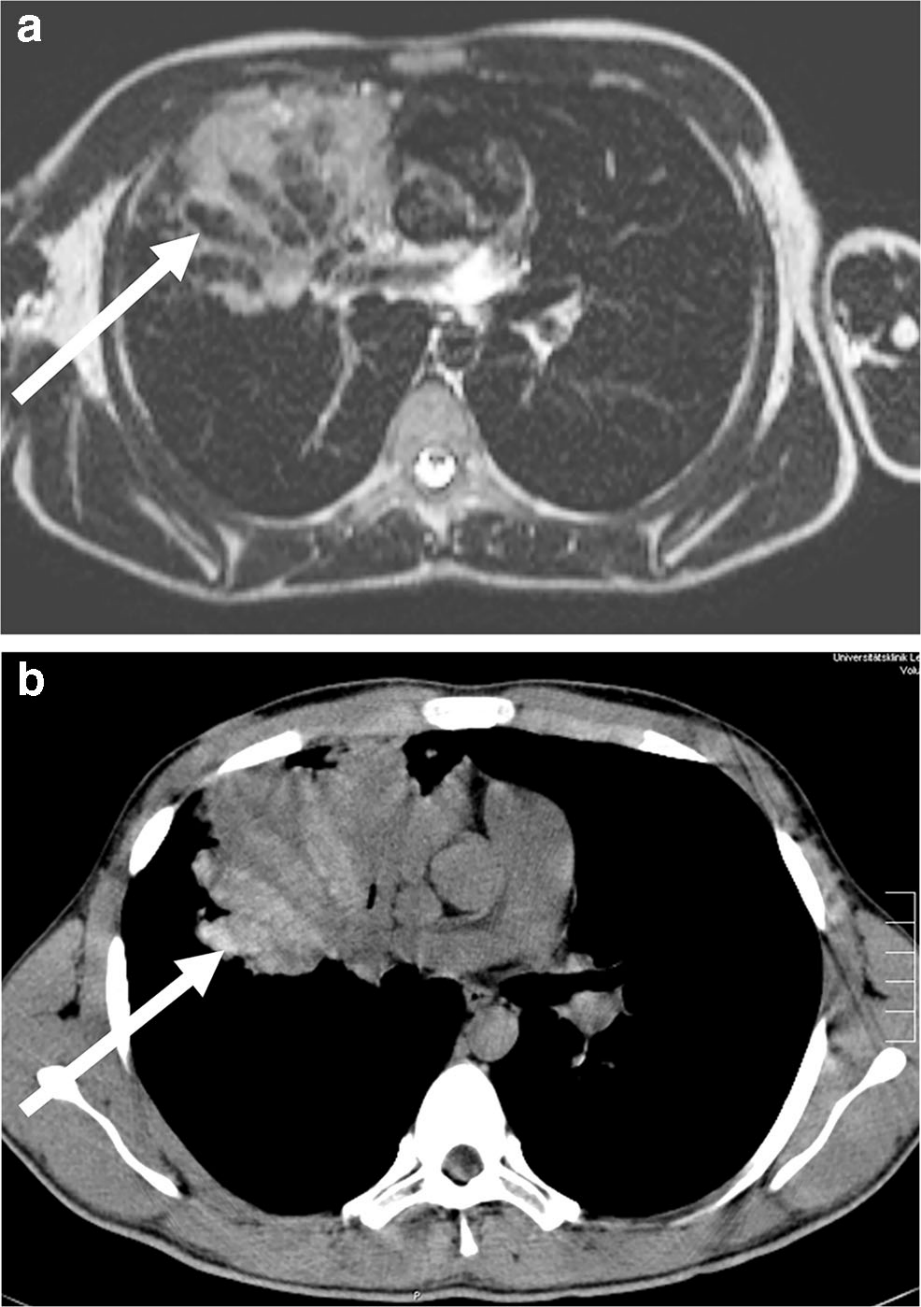
Lung MR in an 11-year-old boy with cystic fibrosis and aspergillomas within bronchiectasis. **a** Axial T2-weighted turbo spin-echo MR image (parameters in Table 2) shows aspergillomas in preformed cavities (*arrow*). They can be diagnosed specifically on MR by the increased iron content. They show low signal at T2 weighting. However, confusion with air in the bronchiectases is possible. **b** A low-dose axial CT image shows that the bronchiectases are filled with echogenic aspergillomas of high density (*arrow*)

The many parallel existing pathologies in cystic fibrosis pose a challenge for MRI diagnostics but are particularly important in pediatric radiology [27]. Alveolar and interstitial changes as well as mucus plugging (Figs. 17 and 18) and complications (Fig. 19) have to be depicted sufficiently. MRI has a disadvantage in cystic lesions such that in individual cases contrast media must be used for clarification.

## Minus-pathologies of lung magnetic resonance imaging

**Fig. 20:**
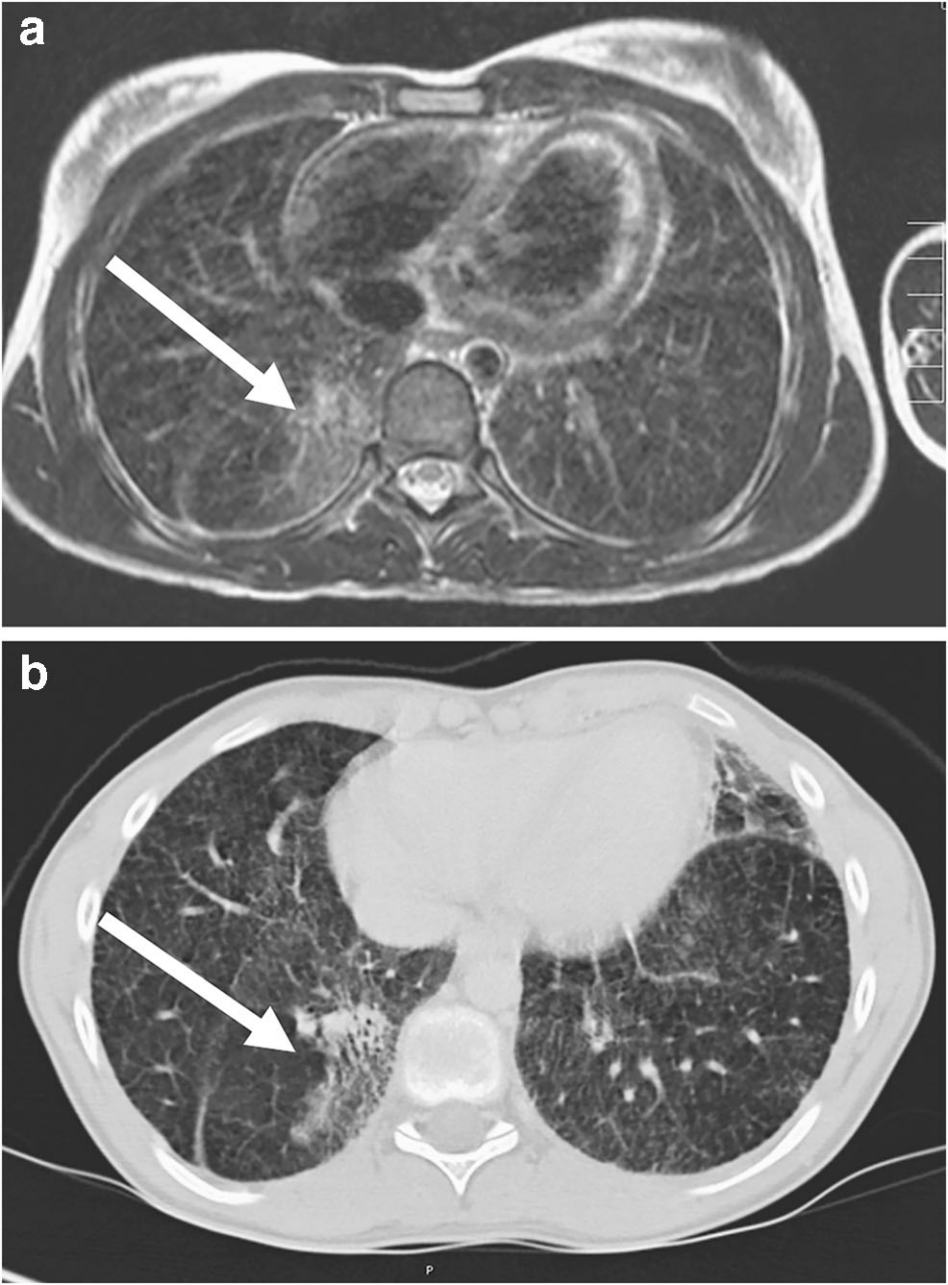
Interstitial lung disease in a 13-year-old girl. **a** Axial T2-weighted turbo spin-echo MR image (parameters in Table 2) shows pronounced interstitial pulmonary fibrosis in the right lower lobe as ground-glass signal increase (*arrow*). **b** On axial CT, interstitial fine granular consolidations can be detected (*arrow*). Fibrosis often cannot be diagnosed on MRI with sufficient certainty

**Fig. 21:**
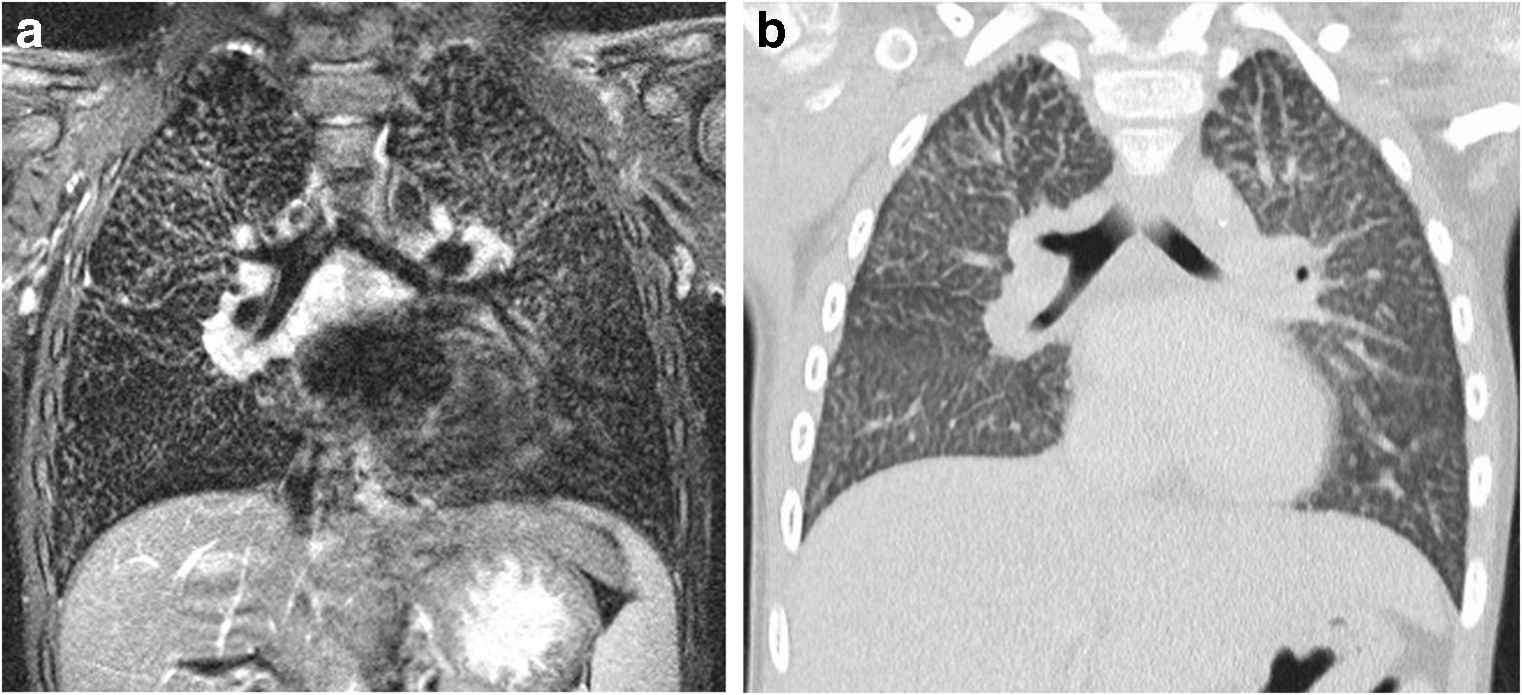
Interstitial lung disease in a 3-year-old boy. **a** Coronal T2-weighted turbo spin-echo MR image (parameters in Table 2) with 3-mm slice thickness at 3 T shows a diffuse ubiquitous fine granular pattern of interstitial lung disease. With a slice thickness of 5 mm, the fine granular interstitial process would hardly be recognizable if it affected the entire lung homogeneously. **b** Corresponding coronal CT image shows similar findings

**Fig. 22:**
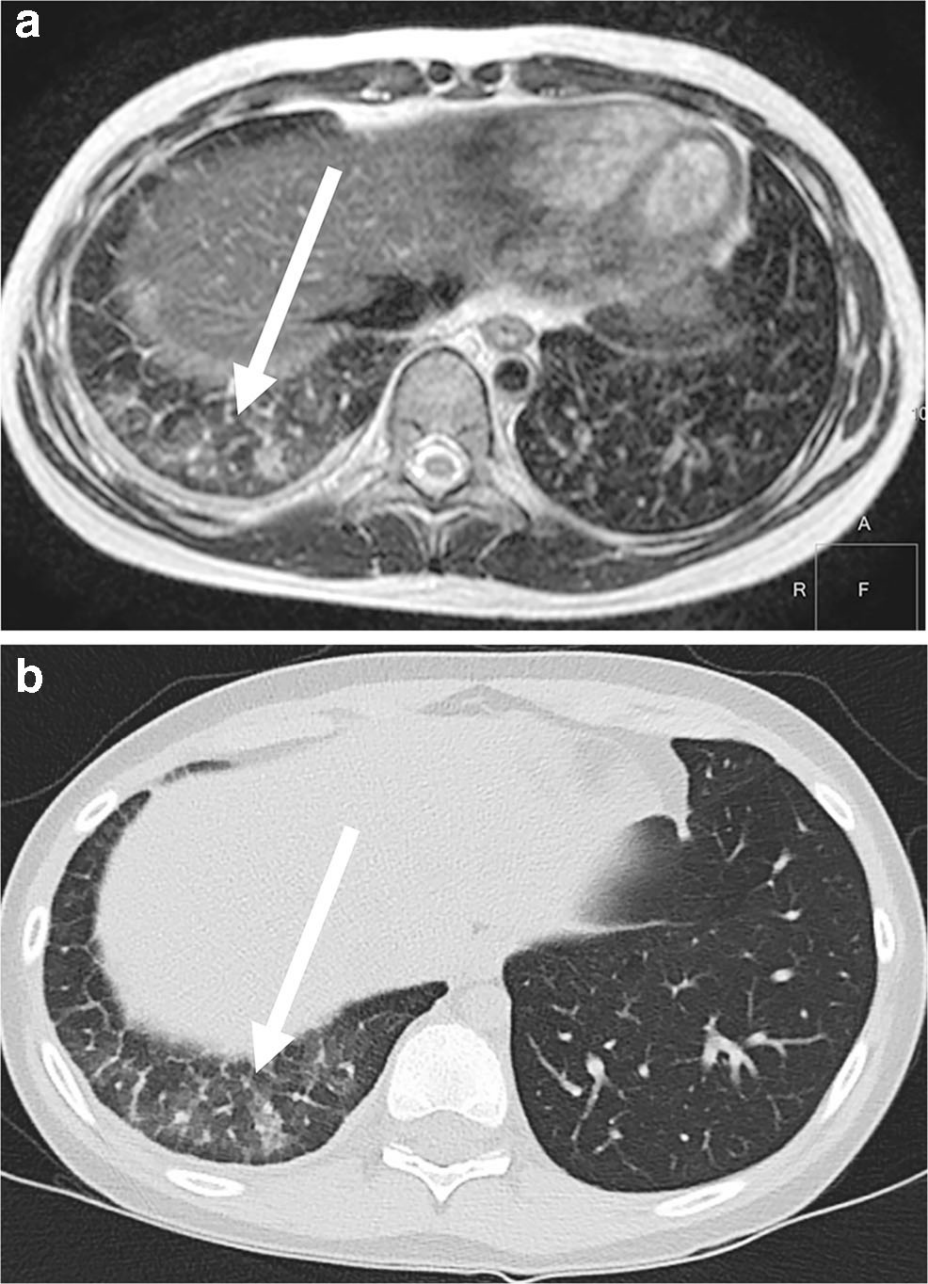
Mycoplasma infection in a 9-year-old boy. **a** Axial T2-weighted turbo spin-echo MR image (parameters in Table 2) shows local interstitial fluid collection from mycoplasma infection and interstitial reaction (*arrow*) in the right dorsal recess. **b** Axial CT shows corresponding findings (*arrow*)

**Fig. 23:**
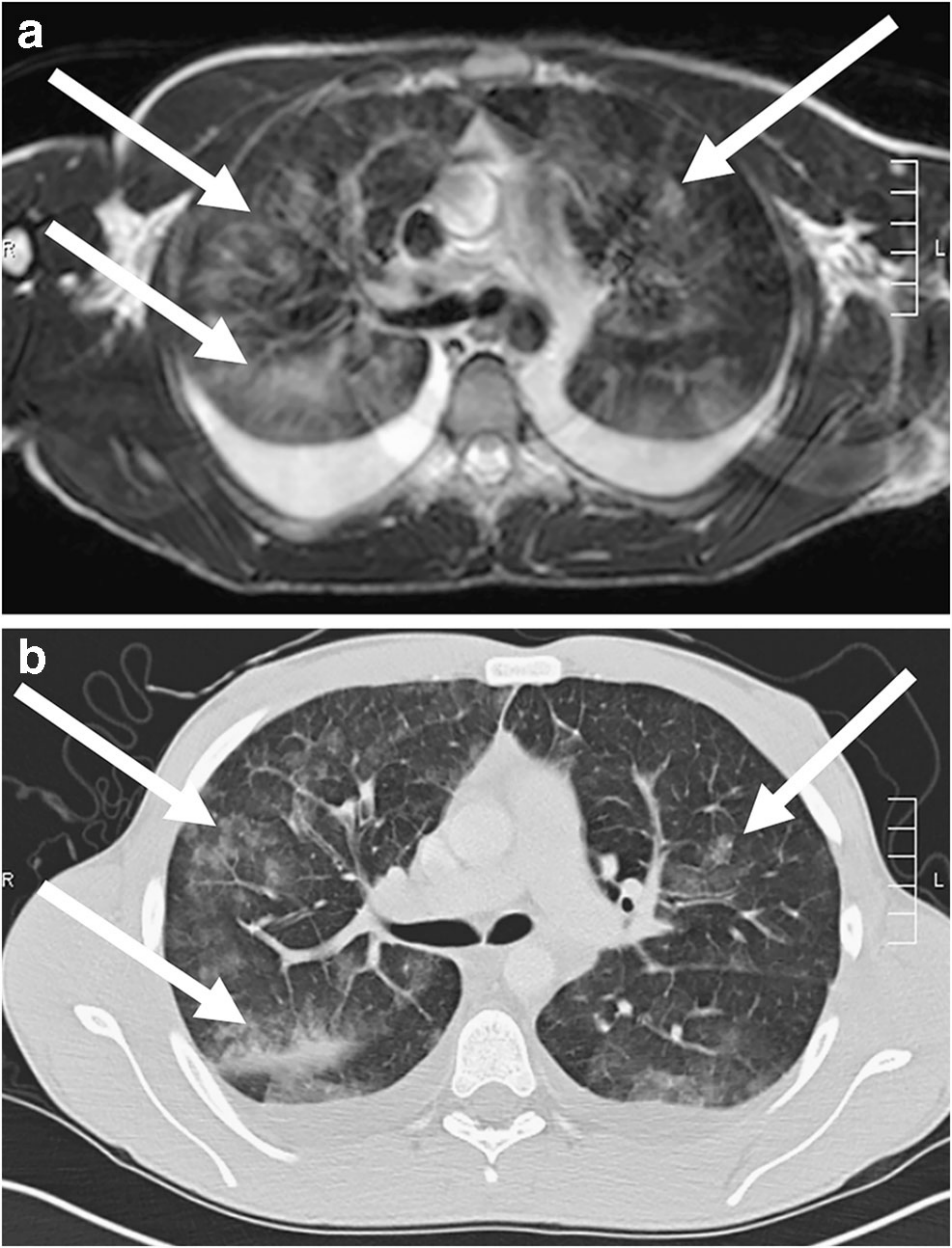
Interstitial edema (*arrows*) caused by lung toxicity in a 7-year-old girl. **a** Axial T2-weighted turbo spin-echo MR image (parameters in Table 2). **b** Corresponding axial CT image

**Fig. 24:**
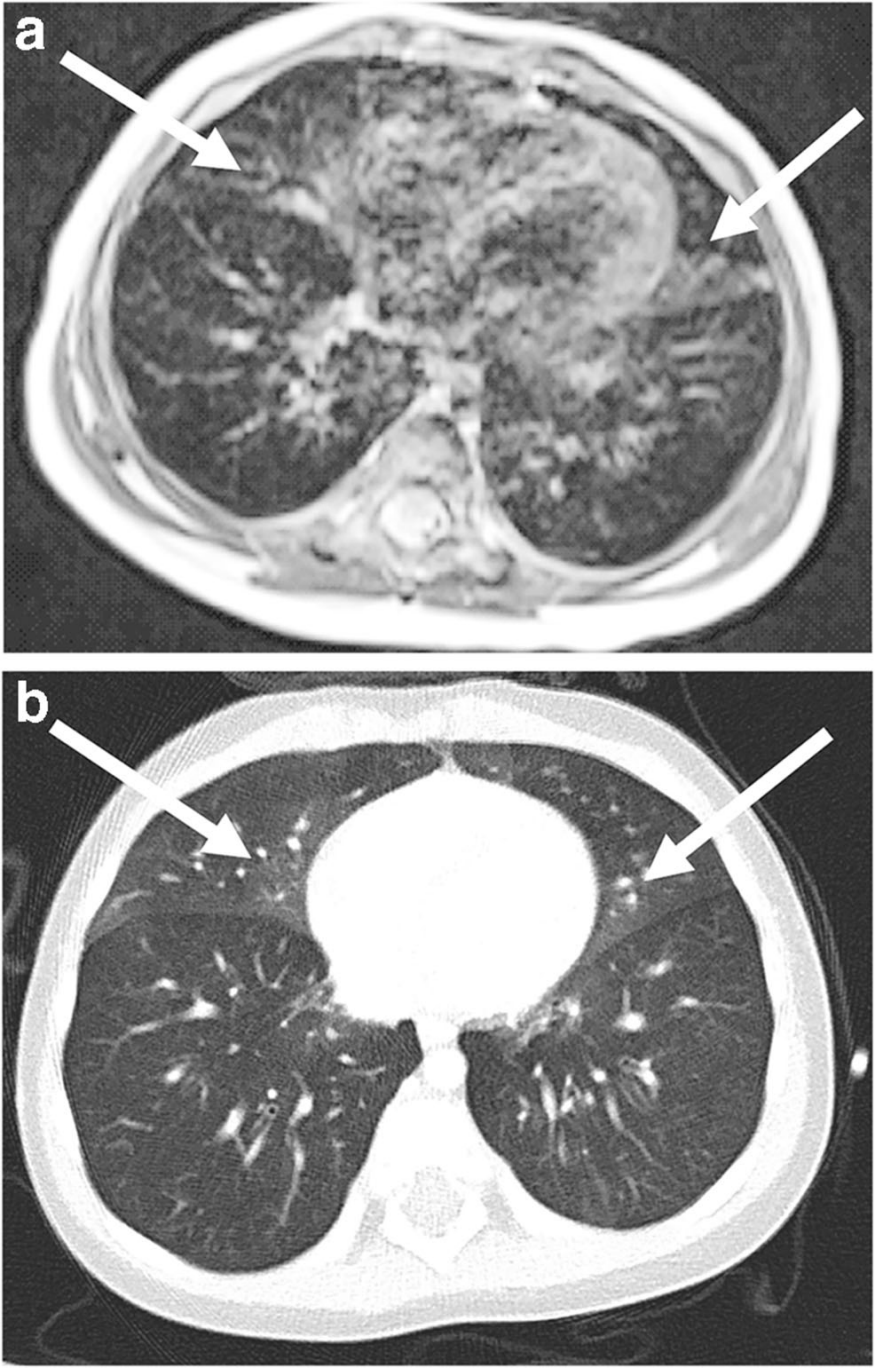
Diagnosis of neuroendocrine cell hyperplasia in an 8-month-old girl. **a** Axial T2-weighted turbo spin-echo MR image (parameters in Table 2) shows multisegmental interstitial ground-glass opacities in the right middle lobe and in Segment 5 of the left lung (*arrows*). The findings are not very pronounced. MR diagnosis of the condition should not yet be recommended in our experience. **b** Corresponding CT image shows subtle ground-glass opacities (*arrows*)

**Fig. 25:**
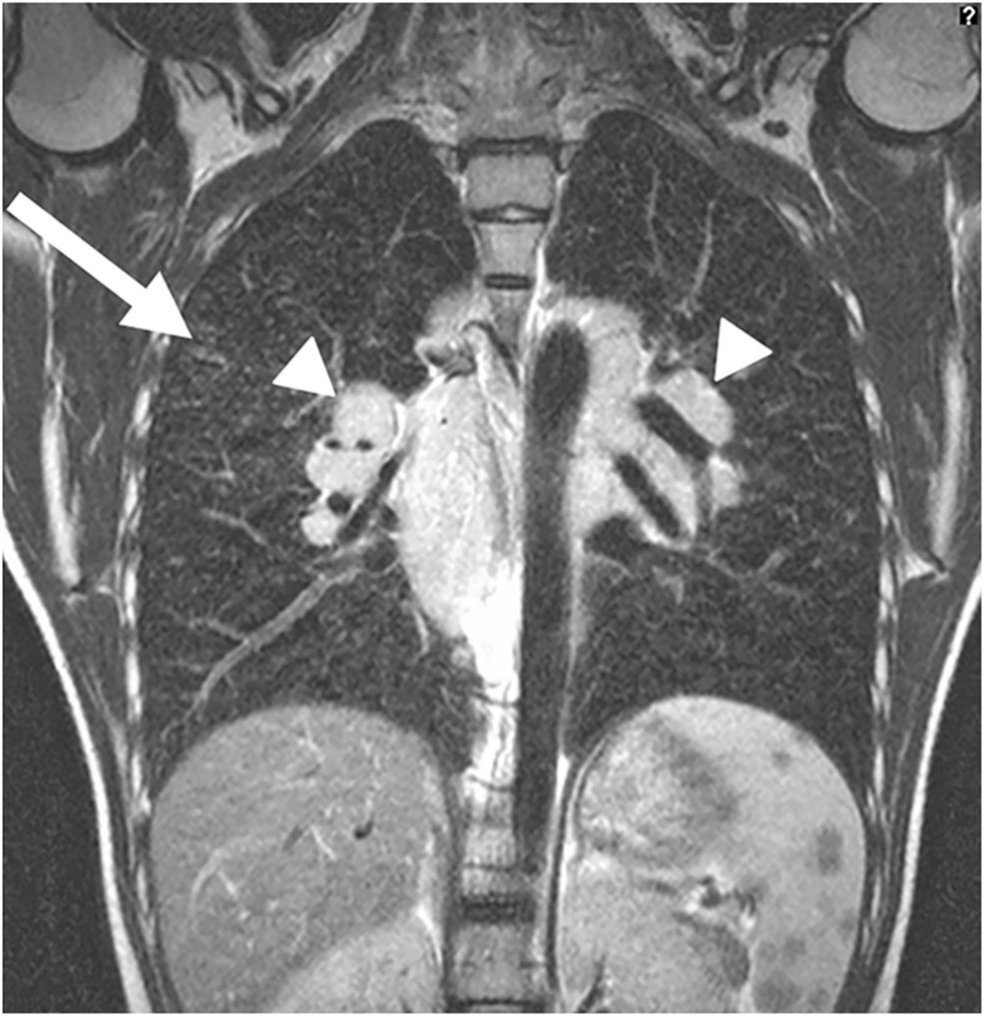
Sarcoidosis Stage II in a 15-year-old girl. Lung MR (coronal T2-weighted turbo spin-echo image, parameters in Table 2) shows many small granulomas, especially in the right lung (*arrow*). This finding, in conjunction with hilar lymph node enlargement (*arrowheads*), is typical for sarcoidosis. A central signal reduction in the lymph nodes, which we could not observe in our patient, is also known to be characteristic. Please also note the low-signal granulomas in the spleen

**Fig. 26:**
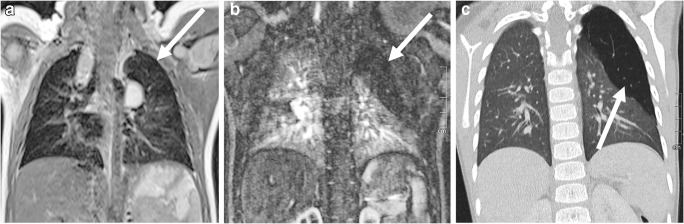
Emphysema of the left upper lobe in a 7-day-old boy on lung MRI (parameters in Table 2). **a** The upper-lobe emphysema is not clearly visible on coronal T2-W turbo spin-echo image (*arrow*). It is caused by a small single thoracic cyst with a diameter of 2 cm. **b** The over-inflation with the resulting capillary reduction (*arrow*) only becomes visible on the time-resolved coronal MR angiogram because of the lack of contrast enhancement of the left upper lobe. **c** Corresponding coronal CT image shows the left upper lobe over-inflation (*arrow*)

The lung diseases in Figs. 20, 21, 22, 23, 24, 25 and 26 represent examples of MR-minus pathologies with regard to the difficult representability on MRI. If fibrosis, cysts, CPAM Types I and II and emphysema are suspected, lung CT might still be indicated as first-line diagnostic test in children [28]. Although MR findings are often very discreet in such MR-minus pathology, MRI can provide helpful diagnostic information in addition to the CT [29].

## What is new about ultrashort echo time sequences?

**Fig. 27:**
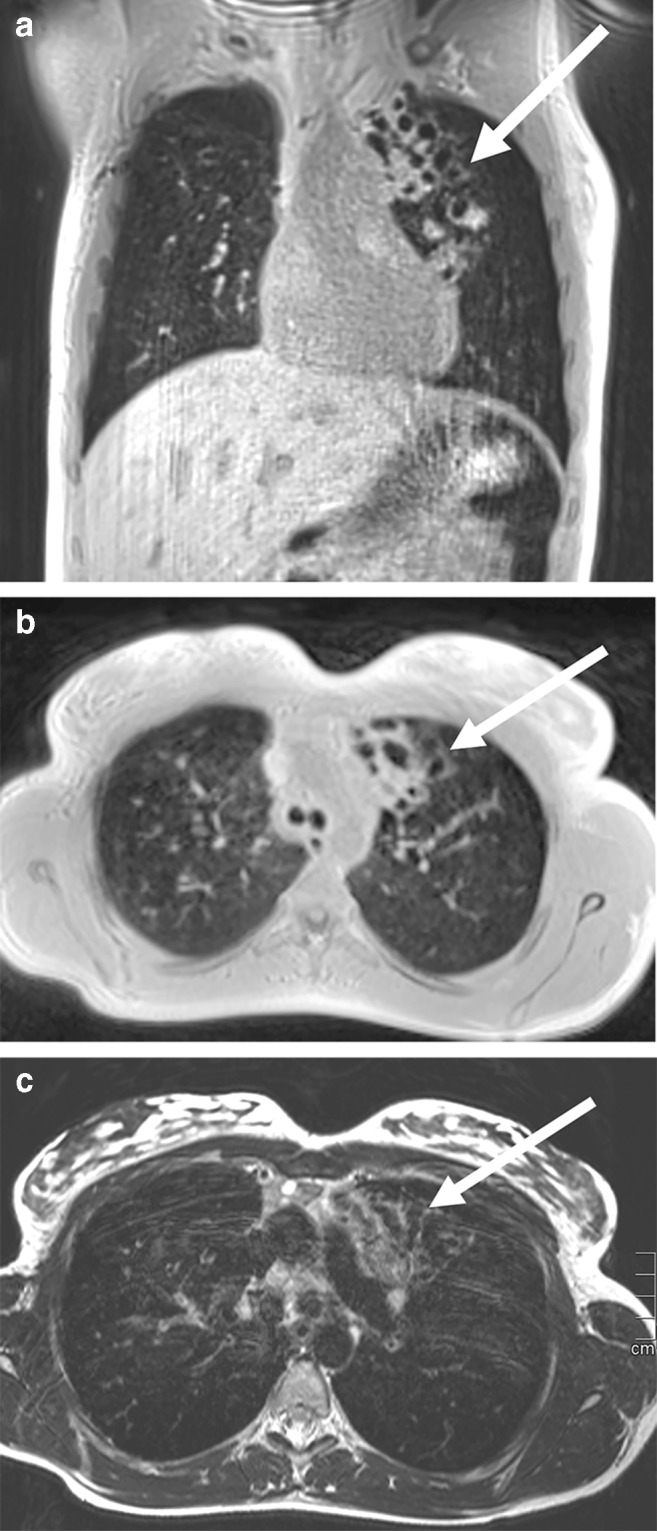
Lung MR in a 15-year-old girl with cystic fibrosis. **a–c** Self-gated T1-weighted ultrashort echo time (UTE) 3-D gradient echo sequence in coronal (**a**) and axial (**b**) planes, and respiratory-triggered axial T2-weighted turbo spin echo (**c**) MR images show bronchiectasis (*arrow*) in the left upper lobe. The CT-like characteristics of the thin slices in the T1-W UTE sequence (0.86-mm isotropic voxels) only become apparent during scrolling. Therefore, movies showing the complete examination sequence are shown in the Online Supplementary Material to this article (Movies 1 and 2)

The use of T1-weighted UTE sequences for lung imaging was described in 2007 by Togao et al. [30]. But only now this idea is being taken up again and technically implemented, after the device manufacturers were able to realize extremely short echo times. Several working groups have since reported on the successful introduction of this 3-D sequence as a non-contrast-enhanced sequence with T1 weighting [31, 32]. The sequences suitable for T1-W 3-D imaging have a high resolution in the submillimeter range and are therefore also suitable for reconstructions in all three planes because of their isovoxel-geometry of 0.86 mm (Fig. 27). The sequence is used without contrast medium. It is self-gated to the respiratory phase and is additionally performed with a special spiral K-space readout. This further reduces movement artifacts [19]. The T1-W UTE sequences are available as 3-D sequences in reconstructible isovoxel geometry or as 2-D sequences, which can be executed in 13 s (slice width 2.5 mm) and also as so-called Zero Echo Time sequences. Technical background of these three lung sequences can be found in an excellent review by Wielpütz et al. 2019 [33].

## Functional magnetic resonance imaging

**Fig. 28:**
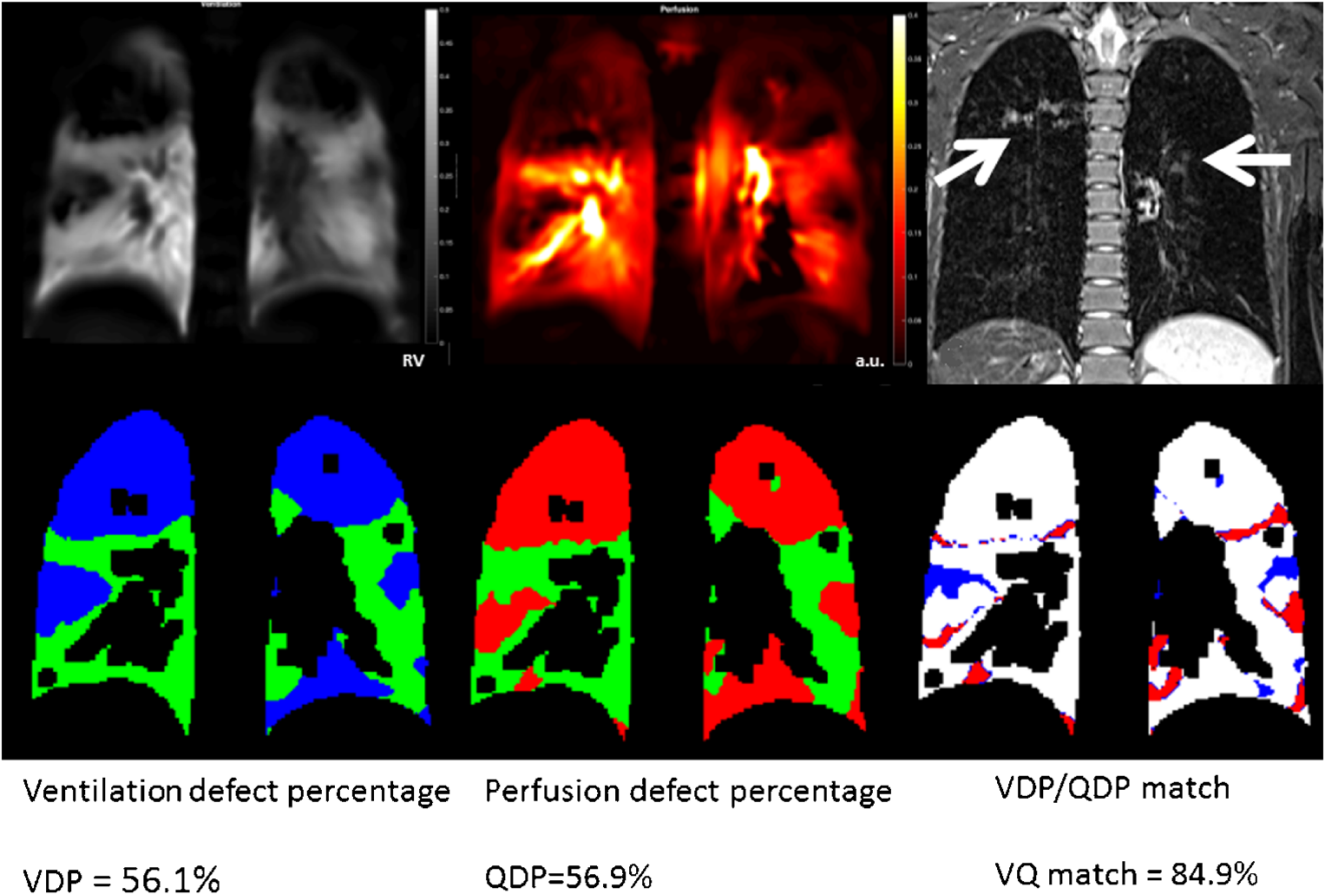
Phase-resolved functional lung (PREFUL) MRI in a 13-year-old girl with cystic fibrosis. Coronal PREFUL MRI-derived regional ventilation (*left column*) and perfusion-weighted image (*middle column*) with ventilation defect percentage (VDP, *blue*), perfusion defect percentage (QDP, *red*) and ventilation/perfusion (VDP/QDP) maps (*white,* signifying matched ventilation-perfusion). The girl has typical heterogeneous areas of hypoperfusion (*red*) and hypoventilation (*blue*), which are predominantly matched on the VDP/QDP map (*white*), mainly because of mucus plugging and bronchial wall thickening in the central upper airways (*arrows*), as shown on the corresponding turbo inversion recovery magnitude image. Movies in the Online Supplementary Material show PREFUL MRI-derived regional ventilation over the whole ventilation cycle (Movie 3) and perfusion-weighted images during the whole cardiac cycle (Movie 4)

Very promising results of dynamic regional ventilation and perfusion mapping using phase-resolved functional lung (PREFUL) MRI (Fig. 28) [4, 34–36], a further development of Fourier decomposition MRI [37], have been shown recently. This technique holds the promise to mature into a patient-friendly MRI spirometry test, with novel clinically relevant information to guide clinical decision-making and improve patient monitoring [38–40]. PREFUL MRI typically uses standard 1.5-T or 3-T MRI equipment and is based on a routine gradient echo fast low-angle shot (FLASH) sequence. PREFUL is well suited especially for children, because it is a free-breathing exam without the need for intravenous contrast agent and has a relatively short examination time (about 1 min per coronal 2-D slice, 15-mm slice thickness). The ventilation, perfusion and dynamic flow-volume loop maps are reconstructed entirely after the image acquisition using complex registration and post-processing algorithms, which are currently available as a research tool.

## Conclusion

The correct indication and thus the preselection of children with a so-called MR-plus pathology are crucial for successful MRI diagnostics of the lungs. T2-weighted turbo spin-echo sequences remain the workhorse of lung imaging in pediatric radiology. However, this only applies if excellent respiratory triggering is achieved, regardless of which of the various trigger methods is used. In the near future, however, T1-weighted UTE sequence is expected to take a dominant position. Because of the current device developments with ultrashort echo times (in the microsecond range), their clinical use is just ahead. The functional evaluations of ventilation and perfusion are also in the preclinical stage and are likely to be an important addition to morphological diagnostics for specific questions.

## Electronic supplementary material

ESM 1 (WMV 4629 kb)

ESM 2 (WMV 4833 kb)

ESM 3 (AVI 1609 kb)

ESM 4 (AVI 1103 kb)
